# Neuroimmune–oligovascular signaling shapes oligodendrocyte precursor cell fate and repair after ischemic stroke

**DOI:** 10.1186/s12964-026-03299-9

**Published:** 2026-10-14

**Authors:** Bin Li, Ruixi Ming, Zijun Wu, Shifeng Pan

**Affiliations:** 1 Institute of Comparative Medicine, Jiangsu Co-innovation Center for Prevention and Control of Important Animal Infectious Diseases and Zoonoses, Yangzhou University, Yangzhou, 225009 Jiangsu China; 2 Molecular Physiology, Center for Integrative Physiology and Molecular Medicine, University of Saarland, 66421 Homburg, Germany

**Keywords:** Ischemic stroke, Oligodendrocyte precursor cells, Cell communication, Signal transduction, Neuroimmune–oligovascular crosstalk

## Abstract

**Background:**

Ischemic stroke causes neuronal and oligodendroglial injury, axonal and myelin damage, and neurovascular unit dysfunction. Oligodendrocyte precursor cells (OPCs) are lifelong progenitors that generate oligodendrocytes while receiving and transmitting signals within neural, immune, and vascular microenvironments. This review examines how neuroimmune–oligovascular communication and intracellular signaling shape OPC survival, recruitment, state transitions, differentiation, and remyelination after ischemic stroke.

**Main body:**

We first define OPC lineage identity, functional heterogeneity, and signaling interfaces with neurons, microglia/macrophages, astrocytes, endothelial cells, and pericytes. We then examine how excitotoxic, metabolic, redox, inflammatory, vascular, extracellular-matrix, and regulated cell-death signals converge on OPCs across lesion compartments and disease phases. Severe core ischemia depletes the progenitor pool, whereas partially perfused peri-infarct tissue permits reactive proliferation and migration. During the subacute phase, intercellular signals within the post-ischemic niche influence whether recruited OPCs adopt inflammatory, angiogenic, differentiation-competent, or arrested states. Long-term repair ultimately depends on lineage completion and stable myelin formation rather than OPC accumulation alone. The strongest causal evidence supports signaling from the surrounding niche as a regulator of OPC differentiation and demonstrates that OPC-derived myelin repair contributes to neuronal preservation and functional recovery. By contrast, evidence that endogenous OPCs themselves substantially regulate post-stroke inflammation, angiogenesis, blood–brain barrier integrity, or synaptic reconstruction remains less complete and often derives from culture, conditioned-medium, pharmacological, or transplantation paradigms. Therapeutic strategies are therefore organized according to the signaling and lineage bottleneck they target, including acute OPC preservation, niche remodeling, lineage completion, oligovascular reconstruction, and cell-based interventions.

**Conclusions:**

OPC fate after ischemic stroke is determined by the interaction of cell-intrinsic signaling competence with temporally and spatially changing neuroimmune–oligovascular cues. Translation will require lineage-resolved causal studies, human spatial and longitudinal validation, signaling-linked target-engagement measures, and treatment strategies matched to lesion phase, compartment, and repair bottleneck. A cell-communication framework may therefore provide a more mechanistically testable basis for OPC-centered white-matter repair after stroke.

## Background

Stroke remains a major cause of death and long-term disability worldwide. The Global Burden of Disease Study 2021 showed that ischemic stroke constitutes the largest proportion of incident strokes and continues to impose substantial mortality and disability burdens across regions [1]. Cerebral arterial occlusion initiates a temporally evolving cascade of energy failure, excitotoxicity, oxidative and mitochondrial stress, neuroinflammation, blood–brain barrier (BBB) disruption, and regulated cell death [2]. Although reperfusion therapy has transformed acute stroke management, persistent disability reflects continuing injury to glial cells, axons, myelin, and microvessels as well as neurons. These interacting neural, immune, and vascular responses shape tissue reconstruction after the acute phase.

White matter integrity is critical for long-term recovery because axonal conduction and metabolic stability depend on intact myelin and coordinated oligodendroglial, neuronal, and vascular interactions. In experimental ischemic stroke, persistent myelin deficits aggravate delayed neuronal loss and neurological impairment, whereas genetic or pharmacological enhancement of oligodendrocyte generation promotes remyelination, neuronal preservation, and functional recovery [3]. These findings position myelin repair as an active determinant of post-stroke recovery rather than a passive consequence of reduced tissue injury.

Oligodendrocyte precursor cells (OPCs) form a resident progenitor population throughout the adult central nervous system and retain the capacity to generate new oligodendrocytes for myelin remodeling and repair [4, 5]. Beyond oligodendrogenesis, OPCs receive neuronal signals, associate closely with cerebral microvessels, and respond to trophic, metabolic, and immune cues. Endothelial trophic signaling can support OPC survival and proliferation, contributing to the concept of an “oligovascular niche” [6]. These properties position OPCs to integrate neural, vascular, and immune signals during tissue injury.

Cerebral ischemia disrupts this cellular niche and produces heterogeneous OPC responses. Some OPCs succumb to metabolic, oxidative, and regulated cell-death pathways, whereas others survive, proliferate, migrate, or enter differentiation programs. Recent evidence identifying perilipin-2-associated OPC ferroptosis as a contributor to myelin injury illustrates that direct OPC loss can itself aggravate white matter pathology [7]. Conversely, increased OPC abundance does not necessarily indicate successful repair, because reactive accumulation may reflect proliferation or recruitment without completion of differentiation into functional myelinating oligodendrocytes.

Human tissue evidence supports this distinction. In post-mortem ischemic stroke tissue, differentiation-committed GPR17⁺/BCAS1⁺ OPCs accumulated in peri-infarct regions, particularly at later stages of lesion development. Their distribution was associated with reactive microglia/macrophages and astrocytes, while transcriptomic analysis associated persistent inflammatory signaling and an inferred reduction in neuron-to-OPC communication with remyelination failure [8]. These observations suggest that, in the human post-ischemic brain, OPC fate is shaped not only by cell-intrinsic differentiation programs but also by the surrounding neuroimmune and glial niche.

Neuroimmune and vascular crosstalk may determine whether post-ischemic OPC responses progress toward repair or chronic dysfunction. Microglia/macrophages, astrocytes, infiltrating immune cells, endothelial cells, pericytes, and neurons can influence OPC survival, migration, and differentiation through soluble mediators, extracellular vesicles, extracellular matrix components, and direct cellular contacts. In a rat model of ischemic stroke, physical exercise increased microglial TREM2 expression and activin A release, thereby promoting OPC differentiation and remyelination through activin receptor signaling [9]. This study provides direct evidence that modifying a non-OPC component of the post-ischemic niche can redirect OPC fate, supporting the view that OPC-centered recovery is a multicellular neuroimmune–vascular process rather than an isolated lineage response.

The therapeutic potential of modulating OPC fate is supported by several experimental studies. Low-intensity pulsed ultrasound reduced acute OPC loss and subsequently promoted oligodendrocyte maturation and remyelination through regulation of IL-17 A/Notch1 signaling in a mouse model of ischemic stroke [10], illustrating that inflammatory signaling can be therapeutically coupled to oligodendroglial repair. Genetic or pharmacological promotion of oligodendrocyte differentiation similarly improved myelin integrity, neuronal survival, and functional recovery after transient cerebral ischemia [3], while transplantation of exogenous OPCs enhanced oligodendrogenesis, neurite growth, synaptogenesis, and behavioral recovery [11]. Nevertheless, translation remains constrained by OPC heterogeneity, uncertain therapeutic windows, model dependence, and limited direct human evidence.

This review examines how cell–cell communication and signal integration govern OPC fate during ischemic brain injury and repair. We consider how neuroimmune, vascular, neuronal, metabolic, and extracellular-matrix signals converge on OPCs across lesion compartments and disease phases to regulate survival, recruitment, state transitions, differentiation, and remyelination. Particular attention is given to communication between OPCs and microglia/macrophages, astrocytes, endothelial cells, pericytes, and neurons, while distinguishing the relatively strong evidence for niche-to-OPC signaling from the more limited evidence that endogenous OPCs directly regulate inflammation, angiogenesis, BBB integrity, or synaptic reconstruction after stroke. We further evaluate therapeutic strategies according to the signaling or lineage transition they target and identify the experimental and translational requirements for establishing clinically meaningful OPC-centered repair.

The literature search was last updated through September 2026 using combinations of oligodendrocyte-lineage terms with ischemic stroke, cerebral ischemia, remyelination, neuroinflammation, neurovascular, and cell-interaction terms. Searches were supplemented by citation chaining and targeted retrieval of relevant primary studies. Original experimental and human studies were prioritized for mechanistic claims, whereas reviews were used mainly for broader context and identification of additional primary literature. Because this is a mechanistic narrative review rather than a systematic review, evidence was evaluated according to biological directness, lineage specificity, model context, and translational relevance.

## OPC identity, plasticity, and signaling interfaces within the neuroimmune–oligovascular niche

Interpreting post-stroke OPC responses requires clear definition of the cell population under study. Establishing lineage identity and anatomical context is therefore essential for distinguishing OPC-specific responses from changes in neighboring cells or more differentiated oligodendroglial populations.

### Lineage identity and functional heterogeneity

#### Lineage identity and homeostatic organization

OPCs, also referred to as NG2 glia in appropriate experimental contexts, are distributed throughout gray and white matter, although their density and proliferative behavior vary across brain regions and with age. OPCs can also retain functional glutamatergic and GABAergic synaptic input even during mitosis [12].

OPC identity should not be assigned on the basis of platelet-derived growth factor receptor-α (PDGFRα) or chondroitin sulfate proteoglycan 4 (CSPG4/NG2) alone. PDGFRα-expressing vascular and leptomeningeal cells and NG2-expressing pericytes are present in the CNS, particularly in perivascular regions. Reliable identification therefore requires integration of morphology, anatomical location, oligodendroglial transcription factors such as OLIG2 and SOX10, and exclusion of vascular-lineage markers. Consistent with this distinction, single-cell RNA sequencing of 5,072 oligodendroglial-lineage cells from ten regions of the juvenile and adult mouse CNS identified a continuous differentiation trajectory with marked regional and maturation-dependent heterogeneity, while also distinguishing a vessel-associated PDGFRα-positive population from bona fide OPCs [13].

In the adult mouse cortex, OPCs maintain a tiled spatial organization through dynamic process extension and self-repulsion. Loss of an OPC through differentiation, death, or experimental ablation prompts neighboring OPCs to extend into the vacant territory, migrate, and proliferate, thereby restoring local cell density [14]. Restoration of OPC number does not, however, establish successful lineage progression. The relatively uniform distribution of OPCs should therefore be viewed as an actively maintained homeostatic state rather than a static anatomical feature.

#### Developmental origin and lifelong lineage dynamics

In the mouse forebrain, OPCs arise in temporally and spatially distinct developmental waves from ventral and dorsal germinal domains. Lineage-tracing and transcriptomic studies indicate that OPCs derived from different embryonic waves become increasingly similar transcriptionally during postnatal development, consistent with partial convergence toward shared cellular states [15]. Developmental origin is nevertheless not completely erased. Selective ablation and replacement experiments showed that ventrally derived oligodendroglial cells can occupy territories normally populated by dorsally derived cells while retaining distinct transcriptional and functional properties that influence myelin organization and adult behavior [16]. Developmental origin should therefore be considered one source of adult OPC heterogeneity rather than a fixed cell-class boundary.

Human OPC development also includes lineage routes that are not fully represented in rodents. In the developing human cortex, EGFR-expressing pre-OPCs can arise from outer radial glial cells before entering the conventional oligodendroglial trajectory [17]. This species-specific developmental feature should be considered when extrapolating rodent OPC biology to the human brain.

**Fig. 1:**
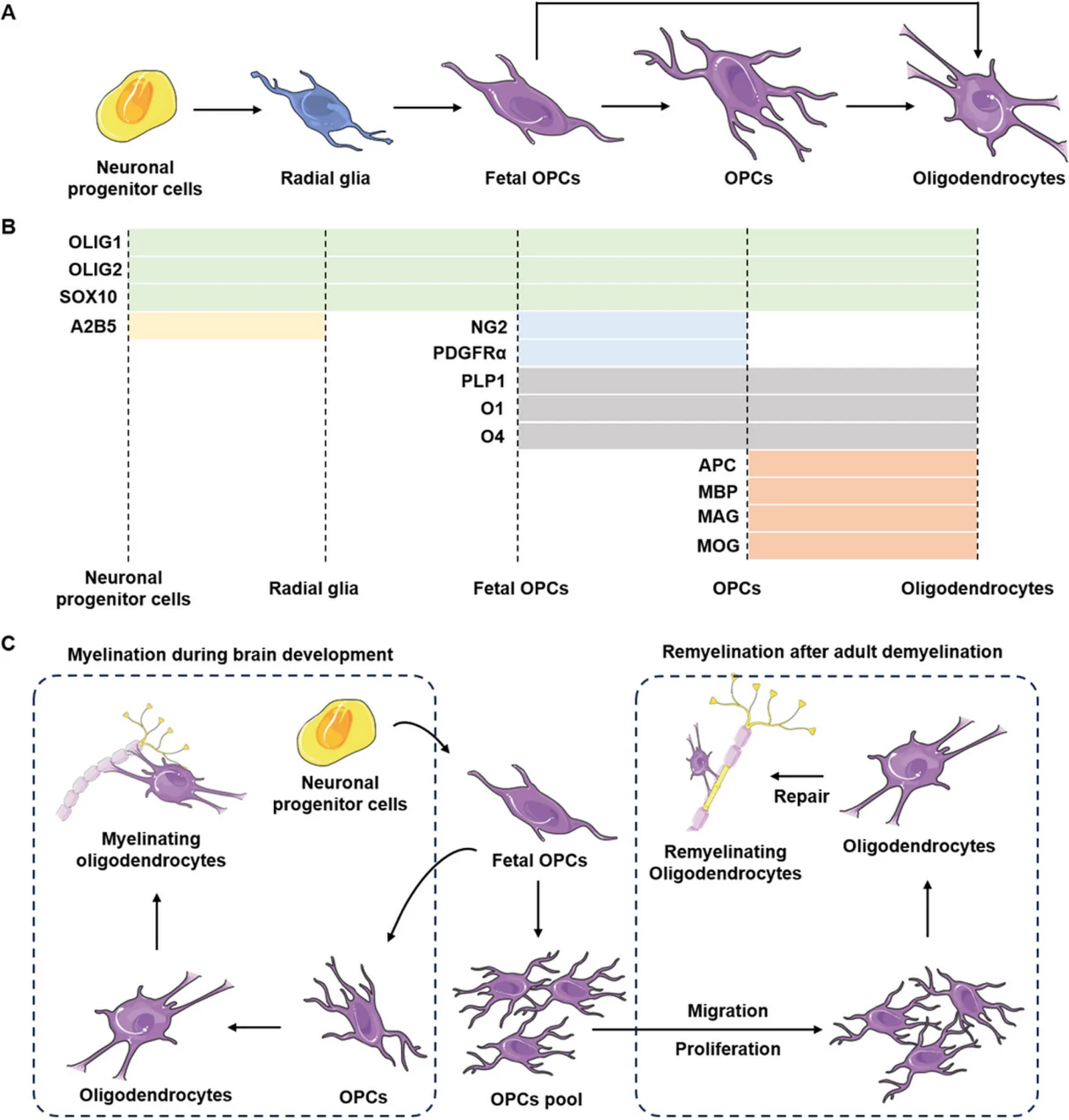
Developmental origin, lineage progression, and lifelong lineage dynamics of oligodendrocyte precursor cells. **A** Neural progenitors and radial glia generate fetal OPCs that enter the postnatal precursor pool and differentiate into oligodendrocytes. **B** Marker enrichment changes across a continuous lineage; no single marker is absolutely stage specific. **C** OPC self-renewal, migration, and differentiation maintain the adult progenitor pool and support developmental myelination and remyelination in experimental injury models. This conceptual diagram is not a quantitative lineage map and should not be interpreted as evidence that adult human oligodendrocyte replacement matches that observed in rodents. Regional heterogeneity, developmental origin, adult homeostasis, and species-related differences are discussed using mouse and human lineage studies [13–18, 20]

Following specification, OPCs progress through overlapping transcriptional states that include proliferative OPCs, differentiation-committed precursors, newly formed oligodendrocytes, and mature myelinating oligodendrocytes. No single marker defines these stages with complete specificity. PDGFRα and CSPG4 are commonly enriched in OPCs; GPR17, BCAS1, and ENPP6 are enriched during lineage commitment and early differentiation; and CNP, MBP, PLP1, MAG, and MOG increase with oligodendrocyte maturation and myelin formation. These lineage transitions, together with developmental origin and maintenance of the adult progenitor pool, are summarized in Fig. 1 [13–17].

Adult oligodendroglial dynamics differ substantially between rodents and humans. Carbon-14 birth-dating studies of the human corpus callosum indicate that oligodendrocyte numbers are largely established during childhood and that oligodendrocyte replacement in adult white matter is limited [18]. Myelin components nevertheless continue to turn over, suggesting that remodeling by existing oligodendrocytes may contribute substantially to adult human myelin plasticity. Robust oligodendrogenesis observed in rodent injury models should therefore not be assumed to reflect the magnitude of lineage renewal available in the adult human brain.

Aging alters both the abundance and functional competence of OPCs. Lifespan transcriptomic profiling in mice showed progressive reductions in OPC proliferation and differentiation together with increased inflammatory, hypoxia-related, and Wnt-associated programs [19]. More recent genetic evidence identified an ACSS2-high OPC population that declines with age; oligodendroglial *Acss2* deletion reduced the OPC pool and impaired myelin maintenance and repair, whereas acetate supplementation improved OPC preservation and remyelination in aged mice [20]. These findings suggest that age-related failure of repair reflects changes in OPC metabolic and transcriptional state, rather than a simple reduction in cell number.

#### Neuron–OPC synapses and activity-dependent lineage responses

Postnatal OPCs receive direct synaptic input from neurons. Glutamatergic and GABAergic signaling can evoke spatially restricted or cell-wide calcium responses, allowing OPCs to couple axonal activity to changes in cellular state [12, 21]. This property is developmentally regulated: proliferative OPCs in the embryonic mouse cortex show little functional synaptic activity, whereas synaptic gene expression becomes prominent after birth and declines again as cells differentiate [22].

The effects of neuronal activity on OPCs are not uniformly proliferative or differentiative. In the mouse corpus callosum, different stimulation frequencies elicited distinct synaptic responses and different effects on OPC proliferation and differentiation, indicating that lineage behavior depends on the temporal pattern of axonal activity [23]. In developing zebrafish, localized synaptic calcium signals predicted sites of subsequent myelin sheath formation, whereas disruption of postsynaptic organization impaired oligodendroglial differentiation and myelination [24]. These findings support activity-dependent control of myelin formation, although evidence from developmental models should not be extrapolated directly to adult ischemic stroke.

Neuron–OPC communication may also be re-established during lesion repair. In a toxin-induced rodent demyelination model, recruited OPCs formed de novo glutamatergic synapses with demyelinated axons. Suppression of axonal activity, vesicular release, or AMPA/kainate receptor signaling increased the accumulation of undifferentiated OPCs while reducing oligodendrocyte differentiation and remyelination [25]. These findings support a role for activity-dependent signaling in lesion repair, but their relevance to ischemic stroke remains uncertain because ischemic tissue is simultaneously exposed to energy failure, excitotoxicity, inflammation, vascular dysfunction, and axonal injury.

#### Context-dependent phagocytic and immune phenotypes

OPCs can engage in material engulfment, although the extent and functional consequences of this behavior depend on developmental stage and tissue context. Ultrastructural studies in the mouse neocortex identified axonal material and lysosomal structures within OPC processes, providing in vivo evidence for axonal engulfment [26]. In primary rat cultures, OPCs internalized myelin-rich, cortical, and astrocytic debris without immediate loss of viability, but this response was accompanied by reduced expression of genes associated with migration and myelination [27]. Debris uptake may therefore represent a context-dependent OPC response rather than an intrinsically reparative function.

A recent developmental study identified an OPC subpopulation involved in CHD8-dependent synaptic phagocytosis during refinement of the mouse retinogeniculate pathway [28]. This finding supports a role for selected OPC populations in circuit remodeling beyond myelin generation, but its developmental context limits direct extrapolation to adult ischemic lesions.

Under inflammatory demyelinating conditions, interferon-γ (IFN-γ) can induce antigen-processing and major histocompatibility complex (MHC) programs in OPCs, and oligodendroglial-lineage cells can become susceptible to cytotoxic T-cell recognition [29]. Most of this evidence derives from inflammatory demyelination models or multiple-sclerosis-related material and does not establish antigen presentation as a constitutive OPC function after ischemic stroke. Immune-like OPC states in the ischemic brain should therefore be regarded as inducible, context-dependent phenotypes until their functional significance is demonstrated directly in stroke models. The implications of these inducible immune phenotypes for interactions with infiltrating lymphocytes, and the distinction between direct OPC responses and immune-mediated niche effects, are considered further in Section "Resident and peripheral immune cells regulate OPC injury and repair".

#### Metabolic regulation of OPC state and lineage competence

The metabolic properties of OPCs should be distinguished from those of mature oligodendrocytes. Direct axonal metabolic support through monocarboxylate transport and lactate delivery is best established for mature oligodendrocytes and should not be generalized to OPCs. By contrast, genetic evidence implicates acetate metabolism in the maintenance of OPC competence: ACSS2-dependent acetate utilization and histone acetylation support the OPC pool and its proliferative capacity [20]. Current evidence therefore supports a role for cellular metabolism in regulating OPC state and lineage output, but does not establish a general OPC-to-neuron lactate shuttle.

OPCs are therefore a heterogeneous and environmentally responsive population whose properties extend beyond oligodendrocyte generation. These differences are particularly relevant after ischemic stroke, where OPCs are exposed simultaneously to altered neuronal activity, vascular dysfunction, inflammatory signals, and extracellular matrix remodeling.

### OPC interactions within the neuroimmune–oligovascular niche

The neurovascular unit (NVU) comprises endothelial cells, mural cells, astrocytes, neurons, microglia, extracellular matrix, and associated glial populations that together regulate BBB function, cerebral perfusion, and tissue homeostasis. OPCs are not constitutive structural components of every cerebral microvessel, but subsets occupy perivascular positions and exchange signals with endothelial cells, pericytes, astrocytes, microglia, and other neighboring cells. These interactions vary with developmental stage, brain region, vascular state, and injury context. The term “oligovascular niche” is therefore best used to describe a dynamic signaling environment in which vascular, glial, and immune cues converge on OPCs, rather than a fixed anatomical unit surrounding all CNS vessels [30–32].

#### Perivascular localization is region- and developmental-stage dependent

During development, OPCs can use the vascular network as a migratory substrate. In the embryonic mouse brain and spinal cord, as well as in the developing human cortex, migrating OPCs have been observed along the abluminal surface of endothelial cells. This association is regulated by endothelial CXCL12 and OPC CXCR4 downstream of Wnt signaling; persistent Wnt–CXCR4 activity can retain OPCs abnormally on vessels and delay differentiation [32]. Vascular association may therefore facilitate OPC dispersal during development but must be appropriately resolved to permit normal lineage progression.

This relationship is not uniform across CNS regions. In developing human and mouse neocortex, OPC abundance correlates spatially and temporally with vessel density, and experimental OPC depletion reduces vascular density [33]. However, live imaging in the same study indicated that OPC migration occurred predominantly along radial glial processes rather than being determined by vascular proximity. These findings differ from earlier descriptions of vascular-guided migration and likely reflect differences in developmental stage, anatomical region, imaging approach, and the OPC populations examined. Vascular association should therefore not be regarded as a universal route of OPC migration.

Reliable identification should combine OPC-enriched markers with oligodendroglial transcription factors such as SOX10 or OLIG2, together with exclusion of endothelial, pericyte, and fibroblast-lineage markers [13, 31].

#### Bidirectional OPC–endothelial signaling in BBB regulation and angiogenesis

Evidence from developmental models indicates that OPCs can influence BBB maturation. OPC-conditioned medium increased claudin-5, occludin, and ZO-1 expression and reduced permeability in cultured brain endothelial monolayers through TGF-β receptor–MEK/ERK signaling. In the same study, neonatal mice with *Pdgfra*-lineage *Tgfb1* deletion developed cerebral hemorrhage and impaired barrier function, supporting an in vivo contribution of OPC-derived TGF-β1 to early BBB development [30]. These findings support developmental OPC–endothelial signaling but do not establish adult OPCs as essential structural components of the mature BBB.

OPC–endothelial signaling also contributes to developmental white-matter vascularization. In developing mouse white matter, stabilization of HIF signaling in oligodendroglial-lineage cells enhanced WNT7A/WNT7B-dependent angiogenesis, whereas loss of oligodendroglial HIF activity impaired vascular development and myelination [34]. OPCs have also been observed in close contact with endothelial tip cells in neonatal mouse, ferret, and human white matter, where Wnt-dependent signaling contributes to vascular growth under developmental hypoxia [35]. These studies establish developmental coupling between oligodendrogenesis and angiogenesis, but do not demonstrate that the same signaling relationships operate unchanged in adult ischemic tissue.

Endothelial cells can in turn provide instructive signals to the oligodendroglial lineage. During mouse embryogenesis, neural progenitor-derived angiopoietin-1 activated endothelial Tie2 signaling and increased endothelial TGF-β1 production, promoting commitment toward the OPC lineage [36]. More recently, endothelial deletion of *Pdcd1* (encoding PD-1) in developing mice caused hypervascularization, abnormal BBB maturation, and impaired oligodendrogenesis. Mechanistically, loss of endothelial PD-1 increased GREM1 secretion, which suppressed BMP/SMAD signaling in OPCs and disrupted subsequent myelination [37]. These findings support a bidirectional relationship in which endothelial cells actively influence oligodendroglial lineage progression rather than serving solely as a vascular substrate.

Oligodendroglial extracellular matrix may also influence vascular barrier properties. Conditional deletion of laminin-γ1 from SOX10-lineage cells disrupted BBB integrity by increasing endothelial transcytosis and reducing MFSD2A expression, while tight-junction ultrastructure remained relatively preserved [38]. Because the manipulation affected the broader oligodendroglial lineage, the phenotype cannot be assigned specifically to OPCs. Nevertheless, the study indicates that oligodendroglial–vascular signaling can regulate BBB function through transcellular as well as paracellular mechanisms.

#### Pericytes and vascular extracellular matrix shape OPC behavior

Pericytes and OPCs occupy adjacent but distinct compartments along the abluminal surface of cerebral vessels. Immunohistochemical and ultrastructural studies have shown close OPC–pericyte apposition across the basal lamina in postnatal and adult rodent white matter, with similar spatial proximity observed in human post-mortem tissue. In culture, pericyte-conditioned medium increased OPC number, whereas OPC-conditioned medium supported pericyte survival or expansion, suggesting bidirectional trophic interactions [31]. These conditioned-medium studies, however, do not identify the dominant signals in vivo or establish a direct role for OPCs in regulating cerebral blood flow.

Pericytes can also support OPC differentiation during repair. In experimental demyelination, pericytes and pericyte-like cells expanded within lesions, and reducing their abundance delayed OPC differentiation. Conversely, pericyte-conditioned medium accelerated OPC differentiation in vitro and enhanced remyelination in an ex vivo model, with laminin-α2 identified as one of the mediators of this effect [39]. These findings support a regenerative role for pericyte-derived signals, although their relevance to ischemic stroke remains to be established.

Matrix effects are context dependent: whereas appropriate matrix cues can support recruitment and lineage progression, persistent association with an abnormal vascular scaffold may restrict OPC dispersal or maturation. Increased accumulation of vessel-associated OPCs after injury should therefore not be interpreted as evidence of successful repair without demonstration of subsequent lineage progression [32, 39].

#### Neuroimmune and vascular signals within the oligovascular niche

Endothelial cells and pericytes do not regulate OPCs in isolation. Astrocytes, microglia, axons, and extracellular matrix contribute additional signals within the perivascular environment. In experimental demyelination, CXCL12 derived from activated astrocytes and microglia promoted CXCR4-dependent OPC maturation and remyelination [40]. Conversely, persistent inflammatory signaling or inhibitory extracellular matrix can maintain recruited OPCs in proliferative or differentiation-arrested states despite adequate cellular recruitment.

Neuron–OPC synaptic signaling should be distinguished from established mechanisms of neurovascular regulation. Although neuronal activity can influence OPC calcium signaling and lineage behavior, direct evidence that OPCs translate synaptic input into BDNF-dependent endothelial nitric oxide signaling or acute changes in local cerebral blood flow remains insufficient.

#### Ischemia reshapes OPC–vascular signaling

OPC–vascular signaling changes substantially under ischemic and hypoxic conditions. In a mouse model of chronic cerebral hypoperfusion, hypoxic OPCs upregulated VEGFA through HIF1α/HIF2α-associated signaling. Conditioned medium from these cells reduced claudin-5 and occludin expression in brain endothelial cells, while BBB leakage preceded overt changes in myelin content or vascular density [41]. In patients with cerebral small-vessel disease, circulating VEGFA was also associated with greater BBB permeability, although its cellular source could not be assigned to OPCs. These findings support a potential contribution of hypoxic OPC signaling to barrier dysfunction but do not establish direct OPC-mediated BBB injury in human acute ischemic stroke.

Chronic ischemia can also convert the vascular surface into an inhibitory environment for OPC lineage progression. In mice subjected to bilateral carotid stenosis, OPCs accumulated abnormally along cerebral vessels despite impaired oligodendrogenesis. Loss of endothelial caveolin-1 increased heat shock protein 90α (HSP90α) secretion, whereas restoration of endothelial caveolin-1 or suppression of HSP90α normalized OPC–vascular interactions, enhanced oligodendrogenesis, and reduced myelin injury [42]. This study provides direct evidence that perivascular OPC accumulation can coexist with defective lineage progression and that endothelial signaling contributes to this dissociation.

Ischemic OPCs may also acquire a pro-angiogenic secretory phenotype. After transient MCAO, perivascular OPC accumulation was accompanied by increased cortical angiogenesis, while oxygen–glucose deprivation (OGD)-conditioned OPCs upregulated VEGFA, angiopoietin-1, and other pro-angiogenic factors. Delayed administration of hypoxic OPC-conditioned medium enhanced angiogenesis and functional recovery [43], and transplantation studies similarly reported reduced BBB leakage or increased angiogenesis and remyelination through Wnt/β-catenin-associated mechanisms [44, 45]. These findings support the angiogenic potential of OPC-derived signals but do not establish that endogenous OPC secretion is sufficient for vascular repair in vivo, because conditioned medium and transplanted cells alter signal concentration, developmental state, and anatomical distribution.

OPC–vascular interactions are therefore highly context dependent. Accordingly, the fate of recruited OPCs after stroke depends not only on their intrinsic lineage competence but also on the metabolic, inflammatory, vascular, and extracellular signals encountered within the lesion environment.

## Convergent ischemic signaling across sequential OPC fate transitions

Post-ischemic OPC repair can be considered as a sequence of biologically distinct but overlapping transitions: preservation of a viable and lineage-competent progenitor pool, proliferation and recruitment of surviving OPCs, differentiation into newly formed oligodendrocytes, oligodendrocyte maturation, and ultimately formation of stable myelin. The signaling mechanisms discussed below are therefore organized according to the principal transition that they constrain rather than as isolated molecular pathways. Acute metabolic, excitotoxic, redox, mitochondrial, and regulated cell-death signals primarily determine whether an adequate OPC pool survives. Subsequent inflammatory, vascular, metabolic, and extracellular-matrix cues influence whether surviving cells proliferate and redistribute or instead progress toward differentiation and maturation. Importantly, these transitions are not interchangeable: preservation or expansion of OPCs does not establish lineage completion, and increased mature-lineage markers do not by themselves demonstrate newly formed functional myelin.

### Injury and survival: preservation of a lineage-competent OPC pool

The first requirement for post-ischemic oligodendroglial repair is preservation of a viable OPC pool with sufficient competence to participate in subsequent lineage progression. Restriction of oxygen and glucose is among the earliest insults encountered by OPCs after ischemia. ATP depletion disrupts ionic homeostasis and promotes membrane depolarization and extracellular glutamate accumulation [2], exposing oligodendroglial lineage cells to combined metabolic and excitotoxic stress. Because adult OPCs constitute the principal proliferative reservoir for oligodendrogenesis, early metabolic injury can reduce not only OPC survival but also the pool of lineage-competent cells available for subsequent white-matter repair [46, 47].

#### Glutamate receptor-mediated OPC injury

Excitotoxicity represents a direct mechanism by which ischemic metabolic failure can compromise OPC survival and reduce the progenitor pool available for subsequent repair.

OPCs express functional ionotropic glutamate receptors and are therefore directly susceptible to ischemic glutamate accumulation. In primary OPC cultures, oxygen–glucose deprivation induces Ca^2+^ influx and cellular injury through Ca^2+^-permeable AMPA/kainate receptors. Repeated sublethal exposure further increases vulnerability by reducing surface GluA2 expression and thereby increasing receptor Ca^2+^ permeability [48]. In adult mouse white-matter slices, AMPA/kainate receptor blockade preserved oligodendrocytes, axonal integrity, and compound action potentials after oxygen–glucose deprivation [49]. This ex vivo evidence supports a broader contribution of glutamatergic signaling to white-matter injury, although the protection observed cannot be assigned specifically to OPCs.

**Fig. 2:**
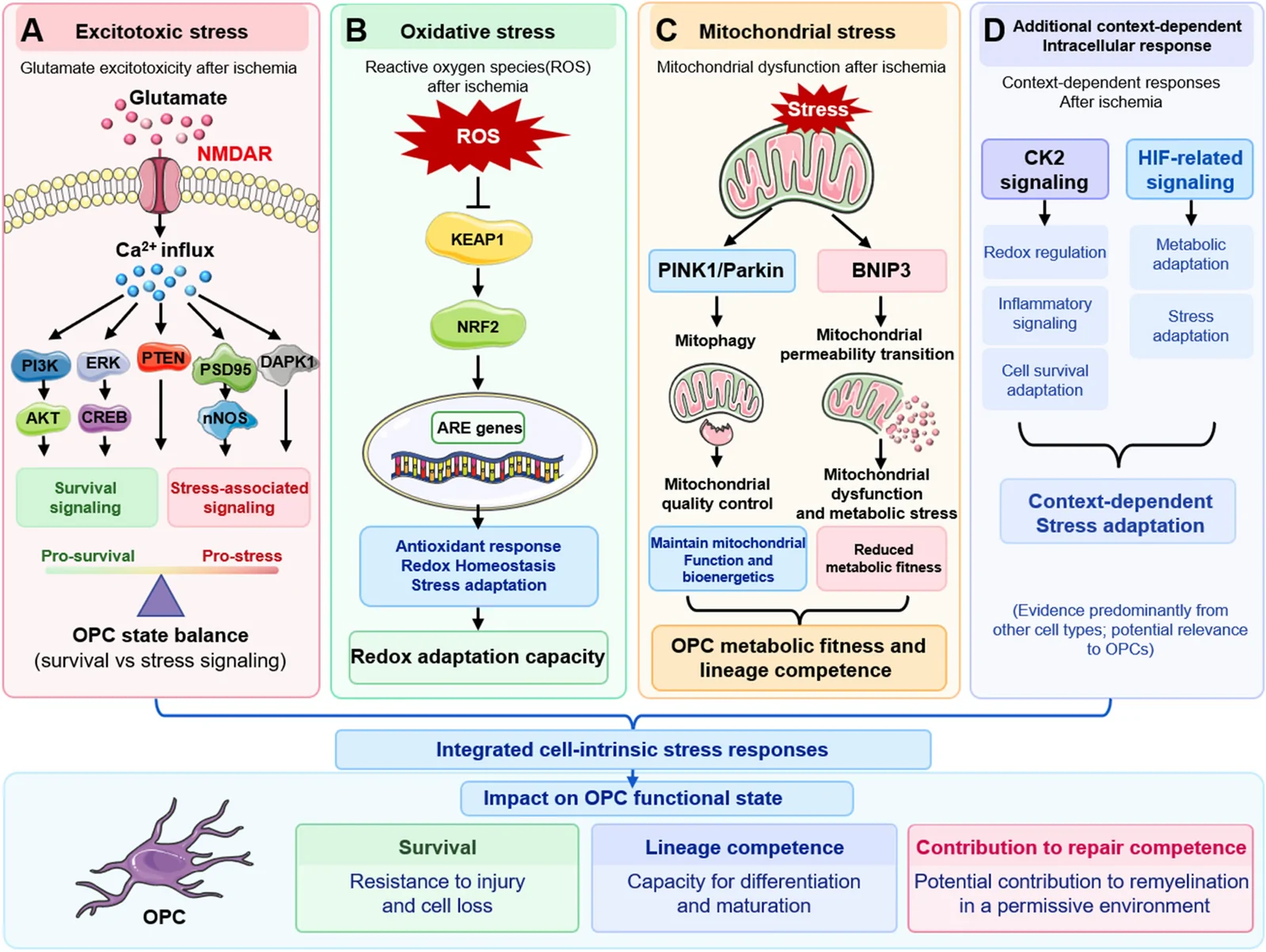
Stress-response pathways relevant to OPC vulnerability and lineage competence after ischemic injury. **A** Glutamate-associated Ca²⁺ overload is presented across different evidence contexts. Ca²⁺-permeable AMPA/kainate receptors have relatively direct support in OPCs, whereas the illustrated NMDAR-associated Ca²⁺ entry and downstream PI3K–Akt/ERK–CREB, PTEN, PSD95–nNOS, and DAPK1 pathways derive mainly from broader oligodendroglial, white-matter, or neuronal studies and are included as mechanistic context rather than as validated adult OPC-intrinsic signaling after ischemic stroke [50–56]. **B** Oxidative modification of Keap1 limits CUL3-dependent Nrf2 degradation, allowing Nrf2 stabilization and antioxidant-response-element-dependent transcription that supports redox adaptation [65–68]. **C** Mitochondrial stress engages both quality-control and dysfunction pathways. PINK1/Parkin-mediated mitophagy is shown as general and neuronal ischemic mitochondrial-quality-control context, whereas BNIP3-associated mitochondrial dysfunction has more direct support in OPCs and premyelinating oligodendroglial cells [59, 73, 74, 76]. **D** HIF-1α-associated OPC responses can bias angiogenic versus oligodendrogenic programs and promote OPC-to-endothelial WNT7A signaling, whereas CK2–JNK/p53 signaling is supported in cultured mature oligodendrocytes and is included only as lineage-stage-specific mechanistic context [69–71]. The figure distinguishes direct OPC or oligodendroglial evidence from pathways included as neuronal, lineage-stage-specific, or general mechanistic context. Pathway arrows summarize the reported direction of signaling and do not imply uniform validation in endogenous adult OPCs after focal ischemic stroke

NMDA receptors may also contribute to ischemic injury within the oligodendrocyte lineage. Simulated ischemia induces glutamate release and activates both NMDA and AMPA/kainate receptors in precursor and mature oligodendrocytes. In white-matter preparations, NMDA receptor blockade, alone or combined with AMPA/kainate receptor inhibition, improved electrophysiological recovery of myelinated axons, although the magnitude of protection varied with the antagonist and experimental conditions [50]. By contrast, downstream NMDAR signaling pathways involving PI3K/Akt–CREB, PTEN, DAPK1, and PSD95–nNOS have been defined predominantly in neurons [51–56]. These pathways are included alongside the more directly supported AMPA/kainate-receptor component in Fig. 2A as mechanistic context for ischemic excitotoxicity, but their operation in OPCs requires direct lineage-specific validation; neuronal GluN2A/GluN2B signaling paradigms should therefore not be assumed to apply unchanged to oligodendroglial cells.

The effects of glutamatergic signaling on OPCs are not uniformly cytotoxic. GluA4 is highly expressed during a developmental stage associated with increased susceptibility to hypoxic–ischemic injury, and NF-Y-dependent regulation of GRIA4 influences both GluA4 expression and oligodendroglial survival under excitotoxic conditions [57]. At lower, subtoxic levels, however, AMPA receptor activation can promote OPC migration through a signaling complex involving αv integrin and proteolipid protein, accompanied by increased intracellular Ca^2+^ transients, reduced extracellular-matrix adhesion, and enhanced cell motility [58]. The consequences of glutamatergic signaling therefore depend on receptor composition, glutamate concentration, exposure duration, and OPC maturation state, which together determine whether receptor activation supports recruitment or shifts cells toward Ca^2+^-dependent injury.

#### Metabolic and mitochondrial constraints on OPC survival

Metabolic failure can injure OPCs independently of glutamate receptor activation. Monocarboxylate transporter 1 (MCT1), expressed prominently within the oligodendrocyte lineage, contributes to lactate transport and intracellular pH regulation. Although oxygen–glucose deprivation can initially increase MCT1 expression, prolonged hypoxia impairs its transport activity, leading to intracellular lactate accumulation and acidification in OPCs. This disturbance was less evident under comparable conditions in neurons expressing predominantly MCT2, suggesting that differences in monocarboxylate transport contribute to the lineage-specific metabolic vulnerability of OPCs [46]. Impaired lactate efflux may therefore restrict the survival of OPCs available for subsequent recruitment and lineage progression.

Mitochondrial stress may further amplify OPC vulnerability during repeated ischemic exposure. In cerebral ischemic preconditioning models, repeated sublethal ischemia stabilized BNIP3 and increased its mitochondrial association, which was accompanied by greater OPC susceptibility to subsequent ischemic injury. BNIP3 silencing or deletion reduced OPC loss and preserved white-matter integrity, while necrostatin-1 also conferred protection [59]. These findings link impaired metabolic adaptation to mitochondrial stress in OPCs, although the specific contribution of BNIP3-associated cell-death signaling is considered separately in Section "Mitochondrial and endoplasmic-reticulum stress-associated cell death".

#### Metabolic adaptation preserves OPC lineage competence

OPCs also possess mechanisms that permit adaptation to reduced glucose availability. Aldolase C (ALDOC), the predominant aldolase isoform in mouse and rat OPCs, is acetylated at lysine 14, a modification that limits activation of the lysosomal glucose-sensing AMPK pathway under low-glucose conditions. Maintaining this attenuated AMPK response was required for appropriate OPC proliferation and differentiation during developmental myelination and remyelination in glucose-poor demyelinated tissue [47]. This mechanism suggests that OPCs can transiently preserve proliferative and lineage-progression programs despite reduced substrate availability rather than mounting a uniformly catabolic energy-stress response.

The relevance of the ALDOC–AMPK checkpoint to ischemic stroke remains uncertain. Its principal in vivo support comes from developmental and experimental demyelination models rather than adult focal cerebral ischemia, and the prolonged or severe substrate deprivation within an infarct may exceed the adaptive range examined in those settings. The pathway should therefore be viewed as a plausible mechanism by which OPCs tolerate glucose-poor environments, rather than as an established determinant of OPC survival or lineage progression after stroke.

Secondary analysis of stroke transcriptomic datasets further suggests that metabolic responses differ across oligodendroglial lineage stages. A 2026 review incorporating re-analysis of three independent mouse stroke single-cell RNA-sequencing datasets identified stage-dependent changes in oxidative phosphorylation, inositol-phosphate metabolism, and sphingolipid metabolism in OPCs, together with increased cholesterol-biosynthetic programs in mature oligodendrocytes [60]. These observations support metabolic heterogeneity across the oligodendrocyte lineage after ischemia, but because they derive from re-analysis of previously published datasets rather than a newly generated experimental cohort, they should be regarded as integrative evidence rather than direct mechanistic validation.

Together, metabolic and excitotoxic mechanisms influence both the size and functional competence of the surviving OPC pool. These effects provide a mechanistic link between early cellular preservation and later lineage progression, but much of the evidence derives from cultures, ex vivo preparations, developmental models, or experimental demyelination rather than adult focal stroke. The following section examines how oxidative and mitochondrial stress further determine whether surviving OPCs retain this reparative competence or progress toward cell death.

### Oxidative and mitochondrial stress in OPCs: from OPC survival to lineage competence

#### Redox injury constrains OPC survival and differentiation

Energy failure and excitotoxic Ca^2+^ loading rapidly converge on oxidative stress during ischemia and early reperfusion. Impaired mitochondrial electron transport and membrane potential increase reactive oxygen species (ROS) generation, to which OPCs appear particularly vulnerable. In primary rat glial cultures, gradual hypoxia produced greater lipid peroxidation and OPC loss than complete anoxia, and OPCs showed a lower capacity for free-radical clearance than astrocytes [61]. These findings identify oxidative injury as a mechanism that can deplete the viable OPC pool before subsequent recruitment and lineage progression occur.

The pentose-phosphate pathway generates nicotinamide adenine dinucleotide phosphate (NADPH) required for regeneration of reduced glutathione and maintenance of cellular antioxidant capacity. In cultured adult rat OPCs and OPC-derived oligodendrocytes, pharmacological NADPH depletion increased superoxide production, reduced glutathione availability, and induced necrotic cell loss. Protection by antioxidants or neurosteroid signaling depended on restoration of reduced glutathione even when NADPH itself was not fully replenished [62]. OPCs also possess cell-intrinsic mechanisms that limit mitochondrial injury. NG2 can bind the mitochondrial serine protease OMI/HtrA2 after its release from damaged mitochondria and restrain its pro-apoptotic activity, whereas genetic or siRNA-mediated reduction of NG2 increases OPC susceptibility to oxidative stress [63]. OPC redox resilience therefore depends on both metabolic antioxidant capacity and intrinsic regulation of mitochondrial death signaling.

Oxidative stress can compromise OPC repair capacity even without causing immediate cell loss. In primary OPC cultures, prolonged sublethal chemical hypoxia suppressed differentiation, whereas the free-radical scavenger edaravone partially restored lineage progression. Similarly, in mice subjected to bilateral carotid artery stenosis, sustained ROS accumulation was accompanied by reduced OPC-to-oligodendrocyte differentiation, myelin loss, and working-memory impairment, all of which were improved by edaravone treatment [64]. Because this model reflects chronic cerebral hypoperfusion rather than focal arterial occlusion, it does not define the acute OPC response to ischemic stroke. Nevertheless, it supports the concept that persistent oxidative stress can leave surviving OPCs functionally incapable of completing effective myelin repair.

#### Adaptive redox and hypoxia signaling preserves OPC lineage competence

Nrf2 links redox sensing to both cellular stress adaptation and oligodendroglial lineage responses. Under basal conditions, Keap1 promotes Cullin-3-dependent ubiquitination and degradation of Nrf2, whereas oxidative modification of Keap1 stabilizes Nrf2 and permits antioxidant-response-element-dependent transcription [65, 66]. This core signaling sequence is summarized in Fig. 2B. In cultured oligodendroglial cells, respiratory-chain inhibition elicited oxidative and integrated stress responses that depended partly on Nrf2 [67]. In mice subjected to brief middle cerebral artery occlusion as an ischemic-preconditioning stimulus, Nrf2 deficiency reduced subsequent OPC generation, migration, and differentiation in the subventricular zone, striatum, and corpus callosum/external capsule [68]. Nrf2 therefore appears to influence both acute stress adaptation and subsequent lineage progression, although its contribution during established focal cerebral ischemia remains incompletely defined.

HIF-1α provides a second link between oxygen availability and OPC functional state. Evidence integrating mouse stroke single-cell datasets with ex vivo hypoxic conditioning and transplantation experiments suggests that the intensity of hypoxia influences OPC phenotype: severe hypoxia favored an angiogenic state, whereas milder hypoxia favored an oligodendrogenic state. Transplantation of severely hypoxia-conditioned OPCs enhanced angiogenesis and functional recovery after transient middle cerebral artery occlusion [69]. These findings support oxygen tension as a determinant of OPC functional adaptation, although the angiogenic and oligodendrogenic states were defined partly through integrated transcriptomic inference and should not be interpreted as fixed or permanently distinct OPC lineages.

More direct evidence identifies a pharmacologically inducible OPC-to-endothelial signaling route after stroke. In a mouse stroke model, oridonin stabilized OPC HIF-1α through PHD2 inhibition, increased perivascular OPC proliferation and WNT7A secretion, and promoted endothelial β-catenin-dependent angiogenesis [70]. These findings identify OPCs as a potential WNT7A-producing cell population and endothelial cells as the downstream vascular recipient, although the study does not establish that the same pathway is constitutively engaged by endogenous OPCs across ischemic conditions.

Together, Nrf2-dependent stress adaptation and context-dependent HIF-1α signaling link early OPC survival to later lineage competence and vascular communication. HIF-1α-associated responses may favor angiogenic or oligodendrogenic states according to oxygen tension and cellular context, but these states should not be treated as fixed lineages or assumed to occur identically in endogenous OPCs after stroke. These context-dependent HIF-1α responses are summarized in Fig. 2D.

#### Lineage-stage-specific redox signaling and metabolic defense

CK2-dependent oxidative-stress signaling has been implicated in cultured mature oligodendrocytes, but its relevance to OPC fate after adult focal ischemic stroke remains unestablished [71]. Accordingly, CK2 is included in Fig. 2D only as lineage-stage-specific mechanistic context, rather than as a validated component of an OPC signaling network.

Redox metabolism also differs substantially across oligodendroglial lineage stages. In a 2026 study using transient middle cerebral artery occlusion and oxygen–glucose deprivation/reoxygenation (OGD/R), phosphoglycerate dehydrogenase (PHGDH), the rate-limiting enzyme of de novo serine synthesis, was induced predominantly in mature oligodendrocytes and astrocytes but was barely detectable in PDGFRα-positive OPCs. Pharmacological PHGDH inhibition reduced glutathione and NADPH availability, increased mature oligodendrocyte death, worsened white-matter integrity, and impaired long-term sensorimotor and cognitive outcomes, whereas supplementation with serine, N-acetylcysteine, or NADPH provided partial rescue [72]. These findings identify serine-dependent one-carbon metabolism as an antioxidant mechanism in mature oligodendrocytes, but not as a directly established protective pathway in OPCs. These differences reinforce the need to distinguish preservation of the OPC progenitor pool from protection of differentiated oligodendrocytes and existing myelin when evaluating subsequent repair.

#### Mitochondrial quality control and antioxidant protection of OPC lineage competence

The preceding evidence indicates that oxidative stress can compromise OPC survival and the differentiation of surviving cells, thereby restricting the cellular basis for subsequent remyelination. Mitochondrial quality-control mechanisms may influence whether such injury is resolved or persists, although their contribution to endogenous OPC repair after adult focal ischemic stroke remains incompletely defined. PINK1/Parkin-dependent mitophagy removes damaged mitochondria through ubiquitin-associated signaling [73], and neuronal ischemia–reperfusion studies have identified PA2G4/EBP1-dependent regulation of this process [74]. These mechanisms are summarized in Fig. 2C, but protective PINK1/Parkin-dependent mitophagy has not been established directly in endogenous adult OPCs after focal ischemic stroke. The more directly supported OPC-associated BNIP3 response is considered separately in Section "Mitochondrial and endoplasmic-reticulum stress-associated cell death".

Antioxidant intervention may also preserve OPC lineage competence while modifying inflammatory signaling. In primary rat OPCs and mature oligodendrocytes exposed to oxygen–glucose deprivation/reoxygenation, vitamin C reduced intracellular ROS and malondialdehyde, attenuated apoptosis, and partially restored OPC differentiation. In OPC–BV2 transwell cultures, vitamin C also reduced pro-inflammatory microglial mediators and increased markers associated with an anti-inflammatory state [75]. These findings link redox protection with OPC–microglial communication, but the study was conducted entirely in vitro with relatively small numbers of biological replicates. It therefore supports mechanistic plausibility rather than therapeutic efficacy in adult focal ischemic stroke.

Severe redox imbalance can drive mitochondrial dysfunction and cell death, whereas sublethal stress may leave OPCs viable but unable to differentiate efficiently or support white-matter renewal. The following section therefore examines how persistent injury can culminate in lineage-stage-specific regulated cell death, depleting either the progenitor pool or the differentiated oligodendrocytes required for myelin maintenance.

### Lineage-stage-specific regulated cell death after cerebral ischemia

**Fig. 3:**
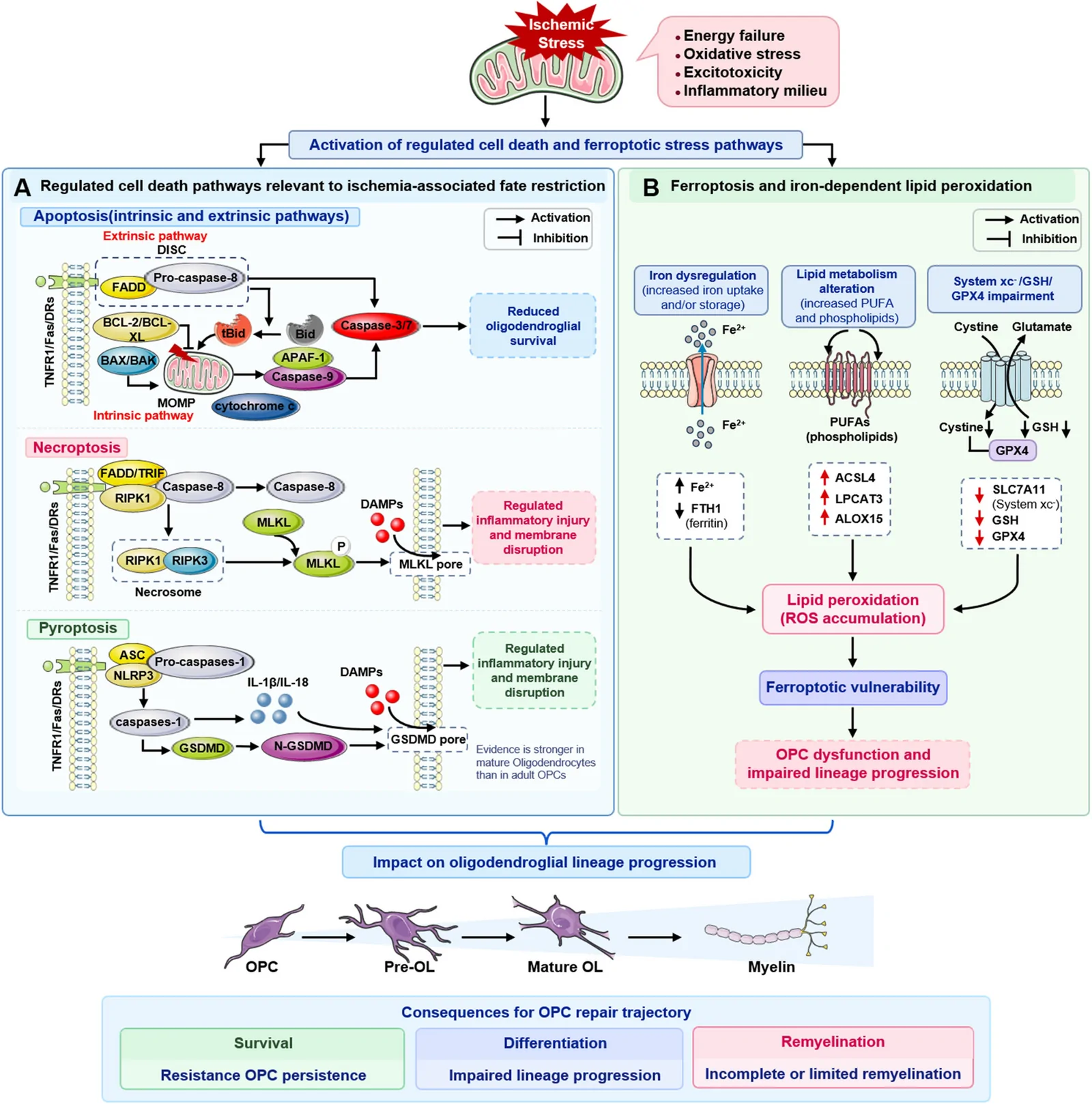
Stress-associated fate restriction and regulated cell-death frameworks across oligodendroglial lineage stages after cerebral ischemia. **A** Regulated cell-death pathways relevant to oligodendroglial injury after cerebral ischemia include apoptosis-, necroptosis-, and pyroptosis-associated signaling, but the strength and lineage specificity of the supporting evidence differ. BNIP3-associated mitochondrial death signaling has relatively direct support in OPCs and premyelinating oligodendroglial cells and is predominantly caspase independent [59, 76]. Canonical mitochondrial and death-receptor-associated apoptosis is shown as general mechanistic context rather than as a uniformly validated pathway in adult OPCs. RIPK1–RIPK3–MLKL necroptosis remains incompletely validated as an OPC-intrinsic pathway [77, 85], whereas ASC–caspase-1–GSDMD-associated pyroptosis is supported mainly in mature oligodendrocytes [78, 83]. **B** Ferroptotic vulnerability reflects the balance between iron-dependent phospholipid peroxidation and system xc−–GSH–GPX4 antioxidant defense. cPLA2α–ACSL4–LPCAT3-mediated phospholipid remodeling and Fenton chemistry are presented as the broader ferroptosis framework. In contrast, peri-infarct OPC loss accompanied by lipid peroxidation and PLIN2 accumulation provides more direct OPC-associated evidence [7], whereas FoxO3a–GPX4 protection is supported mainly by mature-oligodendrocyte-associated and pharmacological readouts [84]. These pathways may overlap, and no single molecular marker is sufficient to establish a specific death mode without complementary morphological, biochemical, and rescue evidence. OPC death, mature-oligodendrocyte loss, and OPC survival with differentiation arrest represent distinct outcomes and should not be interpreted interchangeably

The metabolic, oxidative, and mitochondrial disturbances described above can culminate in multiple, partially overlapping forms of regulated cell death. Their consequences for repair depend on the affected lineage stage: OPC death depletes the progenitor pool available for subsequent recruitment and differentiation, whereas loss of differentiated oligodendrocytes compromises existing myelin and the cells responsible for its maintenance. The relative contribution of individual death programs varies across proliferating OPCs, differentiation-committed precursors, and mature oligodendrocytes, as well as with injury severity and experimental context [7, 76–78]. Evidence obtained in mature oligodendrocytes, neurons, or whole-brain tissue should therefore not be interpreted as direct evidence for the same death mechanism in OPCs. These lineage-stage-specific death frameworks and their principal evidence boundaries are summarized in Fig. 3.

#### Mitochondrial and endoplasmic-reticulum stress-associated cell death

BNIP3 provides relatively direct evidence linking mitochondrial stress to OPC-lineage cell death after ischemic injury. In primary OPC cultures exposed to oxygen–glucose deprivation, BNIP3 accumulation accompanied cell death, while genetic deletion of *Bnip3* reduced pre-oligodendrocyte loss in experimental ischemia. This death response was reported to be predominantly caspase independent and should therefore not be equated with conventional caspase-dependent apoptosis [76]. Repeated sublethal ischemia further stabilized mitochondria-associated BNIP3 and increased PDGFRα-positive OPC loss after a subsequent ischemic insult, whereas BNIP3 knockdown or deletion preserved OPCs and white-matter integrity [59]. These findings support BNIP3-associated mitochondrial death signaling as an OPC-relevant mechanism and indicate that ischemic preconditioning can become maladaptive when mitochondrial stress exceeds OPC adaptive capacity.

Evidence for a causal endoplasmic-reticulum stress pathway in OPC death is less direct. In rat OPCs, ischemia-like insults increased GRP78/BiP, CHOP, and spliced XBP1, but partial XBP1 knockdown did not alter viability after chemical hypoxia or oxygen–glucose–nutrient deprivation, indicating that pathway activation alone does not establish dependence of OPC death on XBP1 [79]. In vivo evidence is stronger for differentiated oligodendrocytes. After MCAO, cytochrome-c release, caspase-12 activation, and TUNEL positivity were associated with demyelination and cognitive impairment and were reduced by AMX0035, although lineage-specific genetic validation was lacking [80]. A separate distal MCAO study identified a non-cell-autonomous injury pathway in which reactive astrocytes produced LCN2, which acted on mature oligodendrocytes through LRP2–JNK3 signaling. Astrocyte-specific *Lcn2* deletion reduced oligodendrocyte loss, secondary demyelination, and cognitive deficits [81]. This establishes an astrocyte-to-mature-oligodendrocyte injury mechanism rather than direct evidence of OPC-intrinsic ER-stress-dependent death. Moreover, protection of existing oligodendrocytes and myelin should be distinguished from generation of new oligodendrocytes or de novo remyelination.

#### Necroptosis: a plausible pathway with limited OPC-specific evidence

Necroptosis is mediated principally through RIPK1–RIPK3–MLKL signaling and contributes to ischemic brain injury, but direct OPC-specific evidence remains limited. In adult male mice subjected to transient MCAO, intracerebroventricular Nec-1 pretreatment preserved OPCs, reduced regional RIPK1, RIPK3, MLKL, and phosphorylated MLKL signals, and improved subsequent white-matter and cognitive outcomes; complementary OPC cultures also showed protection after OGD [77]. However, the intervention was prophylactic and pharmacological, and the in vivo pathway measurements were not lineage specific. These findings therefore support involvement of necroptosis-associated signaling in OPC loss but do not establish an OPC-intrinsic RIPK1–RIPK3–MLKL death pathway genetically. A frequently cited neonatal hypoxia–ischemia study reporting RIPK3–MLKL-mediated oligodendroglial death was subsequently retracted and should not be considered supporting evidence [82]. Accordingly, Fig. 3A presents necroptosis as a mechanistic framework with incomplete OPC-specific validation rather than as an established pathway of post-ischemic OPC death.

#### Pyroptosis and inflammatory propagation of oligodendrocyte loss

Pyroptosis can couple oligodendroglial cell loss to inflammatory amplification, but the strongest post-stroke evidence currently concerns mature oligodendrocytes rather than OPCs. In a 2026 distal MCAO study, astrocyte-derived LCN2 was associated with ASC, caspase-1, and GSDMD activation in oligodendrocytes of the contralateral corpus callosum. LCN2 neutralization or deletion reduced oligodendrocyte pyroptosis, preserved myelin, and improved cognitive performance, whereas astrocytic LCN2 re-expression reversed these benefits [78]. Together with the LCN2–LRP2–JNK3 injury pathway described in Section "Mitochondrial and endoplasmic-reticulum stress-associated cell death", these findings support astrocyte as a source of injury-associated LCN2 and mature oligodendrocytes as the principal demonstrated recipient population. The resulting protection of differentiated oligodendrocytes and existing myelin should not be interpreted as evidence of OPC-intrinsic pyroptosis or de novo remyelination.

Additional evidence for post-stroke oligodendrocyte pyroptosis comes from a 2026 MCAO study in which melatonin reduced the numbers of adenomatous polyposis coli (APC)/caspase-1 and APC/GSDMD double-positive oligodendrocytes, altered microglial inflammatory states, and improved white-matter and sensorimotor outcomes through a RORα/AMPKα/STAT1-associated mechanism [83]. However, the pharmacological design does not establish that functional recovery resulted specifically from inhibition of oligodendrocyte pyroptosis. Moreover, APC identifies differentiated oligodendrocytes rather than PDGFRα-positive OPCs. Adult OPC-intrinsic pyroptosis therefore remains unproven and requires direct lineage-specific validation.

#### Ferroptosis as an emerging OPC-specific mechanism

Among the regulated death programs discussed here, ferroptosis currently has some of the most direct evidence in OPCs after focal cerebral ischemia. This iron- and lipid-peroxidation-dependent form of cell death is promoted when phospholipid oxidation exceeds the antioxidant capacity of the GSH–GPX4 system. In a mouse cerebral ischemia/reperfusion model, peri-infarct OPC loss was accompanied by lipid peroxidation and PLIN2 accumulation in NG2-positive cells. Ferrostatin-1 reduced OPC loss and subsequent myelin injury, while *Plin2* knockdown attenuated demyelination and improved neurological outcomes [7]. These pharmacological, histological, and gene-knockdown data support PLIN2-associated ferroptotic injury in OPCs, although PLIN2 accumulation itself should not be interpreted as proof that every PLIN2-positive OPC undergoes ferroptosis.

Additional MCAO and oligodendrocyte-culture studies have associated increased GPX4 activity with reduced lipid ROS, improved oligodendrocyte survival, and enhanced remyelination [84]. Because these effects were observed mainly in mature oligodendrocyte-associated readouts following treatment with a multicomponent herbal formulation, they support a potential FoxO3a–GPX4 antioxidant defense mechanism but do not provide direct OPC-specific evidence. The relationship between GPX4-dependent antioxidant defense and ischemic lipid peroxidation is summarized in Fig. 3B.

#### Interactions among death programs and implications for repair

Apoptosis, necroptosis, pyroptosis, and ferroptosis are experimentally distinguishable but may converge in ischemic white matter. Mitochondrial oxidative stress, inflammatory signaling, and impaired antioxidant defense can engage overlapping death programs, while lytic cell death may further amplify microglial and astrocytic responses [7, 85, 86]. Accordingly, no single marker—including TUNEL positivity, cleaved caspase-3, GSDMD, phosphorylated MLKL, or reduced GPX4—is sufficient to assign a specific death mode without complementary morphological, biochemical, and rescue evidence.

Loss of mature oligodendrocytes directly compromises existing myelin, whereas OPC death reduces the progenitor pool available for subsequent repair. A third outcome is equally important: OPCs also survive ischemic injury yet remain differentiation arrested, preserving cell number without restoring lineage competence or remyelination. Representative evidence for the major death programs and their principal limitations is summarized in Table 1. The following section examines how inflammatory, vascular, and other niche-derived signals influence whether the surviving OPC pool proceeds through recruitment, differentiation, and maturation toward effective white-matter repair.

**Table 1:** Representative evidence defining OPC fate under ischemic or ischemia-related conditions

Mechanism or question	Target/readout	Model or material	Principal inference	Key reference
Glutamate excitotoxicity	OPCs, oligodendrocyte survival, and white-matter function	Primary OPC cultures; adult white-matter slices	AMPA/kainate receptor activation contributes to Ca²⁺ loading, oligodendroglial injury, and conduction failure	[48, 49]
	Main boundary: Not direct in vivo evidence from adult focal stroke			
MCT1-dependent metabolic stress	OPC lactate transport and intracellular pH	Cultured OPCs under persistent hypoxia	Impaired lactate efflux and acidification contribute to OPC vulnerability	[46]
	Main boundary: Causal importance in adult stroke remains untested			
Endoplasmic-reticulum stress	OPC stress markers and viability	Rat OPC chemical hypoxia and oxygen–glucose–nutrient deprivation	Ischemia-like stress activates GRP78/BiP, CHOP, and spliced XBP1	[79]
	Main boundary: Partial XBP1 knockdown did not improve survival; activation is not proof of causal dependence			
ALDOC–AMPK glucose sensing	OPC proliferation and differentiation	Developmental and demyelination models	OPCs use a lineage-specific low-glucose checkpoint	[47]
	Main boundary: Mechanistically relevant but not established in focal stroke			
Hypoxia-directed state transition	Angiogenic versus oligodendrogenic OPC states	Mouse tMCAO; OPC secretome; integrated datasets; transplantation	Ischemia and oxygen severity influence vascular versus oligodendrogenic OPC programs	[43, 69]
	Main boundary: Conditioned medium, transcriptomic inference, and transplanted cells do not prove endogenous sufficiency			
PLIN2-associated ferroptosis	Peri-infarct NG2⁺ cells and myelin	Mouse cerebral ischemia/reperfusion; ferrostatin-1; *Plin2* knockdown	Direct support for OPC ferroptosis contributing to later myelin injury	[7]
	Main boundary: Requires replication and lineage-specific genetic confirmation			
TNF-α–PAD4 differentiation block	OPC differentiation and white-matter repair	Hyperhomocysteinaemic MCAO and OPC OGD/R	Inflammation can impose an epigenetic block on OPC maturation	[90]
	Main boundary: Applies directly to a defined vascular-risk context			
Microglial TNFR2	Debris clearance and oligodendrocyte maturation	Mouse photothrombotic stroke with conditional manipulation	Microglial repair functions regulate white-matter recovery	[95]
	Main boundary: Marked sex dependence and model specificity			
Pharmacological evidence for necroptosis	OPC survival and regional RIPK1–RIPK3–MLKL signals	Nec-1 pretreatment before mouse tMCAO; OPC OGD	Nec-1 preserves OPCs and white-matter outcomes	[77]
	Main boundary: Prophylactic drug design and non-lineage-specific in vivo pathway readouts			
Human differentiation-committed OPCs	BCAS1⁺ and GPR17⁺ peri-infarct cells	Post-mortem ischemic lesions	Accumulation of early oligodendroglial states without a proportional increase in later maturation markers	[8, 111]
	Main boundary: Cross-sectional tissue cannot establish lineage flux or causal signaling			
Early human lineage-state shift	OPC and mature oligodendrocyte nuclei	Peri-infarct cortex from 2 stroke cases and 3 trauma comparators	Exploratory evidence for early-state OPC enrichment after stroke	[115]
	Main boundary: Very small cohort and non-neurologically normal comparator tissue			

### Niche regulation of OPC recruitment, differentiation, and maturation

**Fig. 4:**
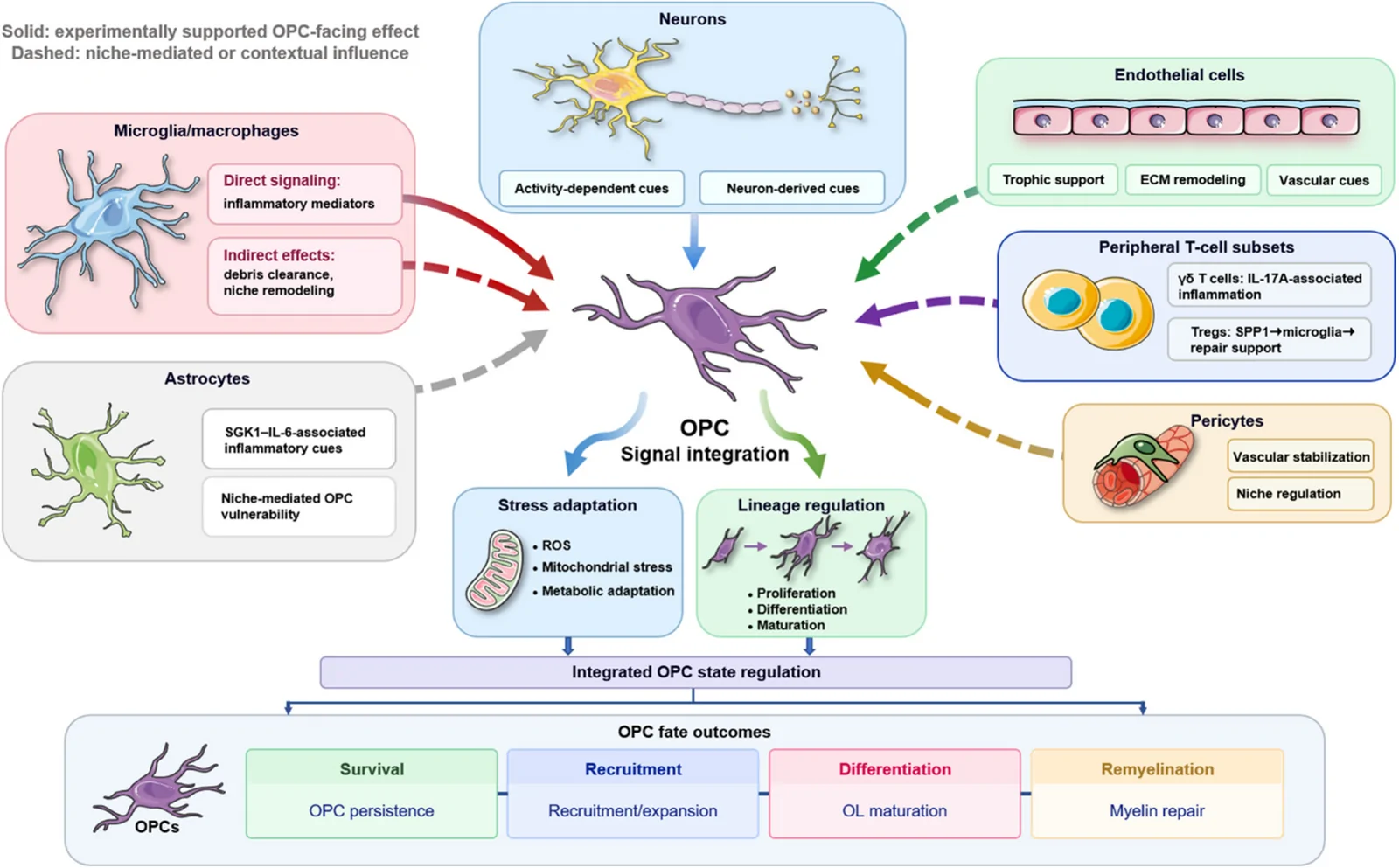
Neuroimmune–oligovascular communication regulates successive OPC fate transitions after ischemic stroke. The post-ischemic niche regulates successive OPC fate transitions through sender-, phase-, and context-dependent communication among resident microglia, infiltrating monocyte-derived macrophages, astrocytes, peripheral T-cell subsets including γδ T cells and regulatory T cells, neurons and axons, endothelial cells, and pericytes. Solid arrows from sender-cell panels to OPCs indicate experimentally supported OPC-facing effects in the cited models; dashed arrows indicate niche-mediated or contextual influences and do not establish direct OPC targeting. Myeloid- and neuron-derived signals can preserve OPC survival or promote recruitment, as illustrated by TGF-α–EGFR–STAT3- and SPP1–CD44-associated routes [88, 89]. During later recovery, microglial TREM2–activin A signaling, regulatory-T-cell–SPP1–microglial communication, TNFR2-dependent debris clearance, extracellular-vesicle cargoes, and macrophage-mediated niche remodeling can support OPC differentiation, oligodendrocyte maturation, or white-matter repair through direct and indirect mechanisms [9, 93–100]. γδ-T-cell-derived IL-17 A-associated inflammation and astrocytic SGK1–IL-6-associated signaling are presented as context-dependent, predominantly niche-mediated influences, whereas their direct control of OPC fate remains incompletely established in adult ischemic stroke [29, 91, 92, 107]. Endothelial, pericyte, extracellular-matrix, and activity-dependent neural cues provide additional context for recruitment and lineage progression [21, 23–25, 31, 32, 36, 37, 39, 40, 42]. These signaling routes converge on distinct checkpoints—OPC survival, recruitment, differentiation, mature oligodendrocyte generation, and remyelination—and should not be interpreted as a single linear pathway. Because the strength, directness, and disease relevance of the evidence differ across adult stroke and non-stroke models, the figure represents an integrated framework rather than a uniformly validated OPC-specific signaling network

Preservation of the viable OPC pool is necessary but insufficient for effective repair. Surviving OPCs must subsequently proliferate or migrate into damaged tissue, acquire differentiation competence, and progress toward mature oligodendrocytes. These transitions are regulated by interactions with resident and infiltrating immune cells, astrocytes, endothelial cells, pericytes, and the extracellular matrix. Inflammatory and vascular signals can support recruitment or lineage progression in one context while promoting injury, abnormal retention, or differentiation arrest in another. This section therefore examines how the post-ischemic niche regulates successive OPC fate transitions, with particular attention to cellular source, disease phase, and the distinction between direct OPC signaling and indirect niche-mediated effects. Fig. 4 integrates the sender–signal–OPC outcome relationships across neural, immune, and vascular compartments, whereas Table 2 summarizes their cellular sources, signaling modalities, OPC-related outcomes, and evidence contexts.

**Table 2:** Neuroimmune–oligovascular signaling modes regulating OPC fate after ischemic stroke

Sender population	Signaling modality	OPC-related outcomes	Evidence context
Microglia/ macrophages	Trophic and inflammatory mediators; extracellular-vesicle cargo; debris clearance and niche remodeling	Context-dependent regulation of OPC survival, recruitment, differentiation, oligodendrocyte maturation, and remyelination	Supported but mechanistically heterogeneous. Stroke and demyelination studies include both direct and niche-mediated effects; cellular source and endogenous necessity are not consistently resolved [9, 88, 89, 93–95, 97–100, 103, 104]
Astrocytes	Inflammatory cytokine signaling; CXCL12/CXCR4-associated signaling; extracellular-matrix and metabolic niche modulation	Context-dependent effects on OPC survival, maturation, differentiation arrest, and remyelination-associated responses	Supported but model dependent. Evidence spans neonatal hypoxic–ischemic injury, experimental demyelination, chronic ischemia, and focal white-matter stroke; direct causality in adult focal stroke remains incomplete [40, 107, 121]
Peripheral T cells/Tregs	IL-17 A- and IFN-γ-associated inflammatory signaling; Treg-derived SPP1 acting indirectly through microglia	Predominantly indirect regulation of OPC vulnerability, oligodendrogenesis, and white-matter repair through inflammatory-niche remodeling	Emerging and context dependent. Direct T-cell-mediated OPC injury has not been established as a major mechanism in adult ischemic stroke; Treg–SPP1 evidence supports an indirect microglial route [29, 91, 92, 94]
Endothelial cells	Vascular trophic signals; extracellular matrix-associated cues; vascular niche regulation	Modulation of OPC maintenance, recruitment, and lineage progression	Supported but context-dependent. Evidence derives largely from developmental and chronic ischemic settings [6, 32, 36, 37, 42]
Pericytes	Trophic support; extracellular matrix and vascular niche modulation	Potential regulation of OPC differentiation and repair-associated states	Emerging. Evidence from experimental repair models; ischemic relevance remains unresolved [31, 39]
Neurons/ axons	Activity-dependent cues; neuron-derived signals	Regulation of OPC calcium signaling, state transition, and lineage behavior	Context-dependent. Evidence supports neuron–OPC interactions, whereas causal links to ischemic remyelination remain insufficiently established [21, 23–25]

#### Resident and peripheral immune cells regulate OPC injury and repair

Ischemic inflammation does not impose a uniformly destructive pressure on the oligodendrocyte lineage. Its effects depend on lesion compartment, disease phase, and the cellular origin and functional state of the immune response. Resident microglia and infiltrating monocyte-derived macrophages (MDMs) represent distinct myeloid compartments, although their activation programs can converge after injury. This distinction is important when interpreting studies based on Iba1, CD11b, CD68, or other shared myeloid markers, which do not establish cellular origin in isolation. TMEM119 and P2RY12 can assist in identifying resident microglia, but their expression changes during activation; lineage tracing or appropriately validated combinations of markers are therefore preferable when assigning source-specific functions [87]. The following evidence is therefore organized according to the OPC transition affected—survival, recruitment, differentiation, or lineage completion—and whether the immune effect is mediated by direct signaling to OPCs or indirectly through remodeling of the post-ischemic niche.

The preservation of a viable OPC pool is an early requirement for subsequent repair and can be influenced by immune-derived trophic signals. In mouse transient MCAO, transforming growth factor-α (TGF-α) expression increased in neurons and microglia/macrophages, while EGFR expression increased in oligodendroglial-lineage cells. TGF-α deficiency exacerbated OPC and oligodendrocyte death and impaired long-term white-matter integrity. Complementary OGD experiments demonstrated direct protection of OPCs and oligodendrocytes through STAT3-associated signaling, without a corresponding promotion of OPC differentiation [88]. Together, these findings support a TGF-α–EGFR–STAT3-associated survival route involving signal-producing neurons and microglia/macrophages and recipient oligodendroglial-lineage cells. However, the highest directly supported OPC outcome is protection from injury rather than enhanced differentiation, and the in vivo myeloid source was not resolved into resident microglia and infiltrating MDMs.

Once a viable progenitor pool is retained, immune-derived signals may influence OPC recruitment into injured tissue. Single-nucleus RNA sequencing in male rodent stroke models identified infarct-restricted proliferating OPCs alongside reactive astrocytes and stroke-associated myeloid populations. Spatial analyses further showed SPP1-expressing myeloid cells in proximity to CD44-positive proliferating OPCs, while SPP1 enhanced OPC migration in vitro [89]. These findings support an SPP1–CD44-associated recruitment mechanism, but they do not distinguish resident microglia from infiltrating MDMs as the relevant in vivo source, establish the necessity of this pathway in vivo, or demonstrate that recruited OPCs subsequently complete differentiation and remyelination.

Recruitment alone, however, does not ensure lineage completion, and systemic vascular risk can impose a differentiation block through inflammatory signaling. In hyperhomocysteinaemic MCAO and OPC OGD/R models, TNF-α promoted PAD4 nuclear translocation and histone H3 citrullination, reduced myelin regulatory factor (*Myrf*) expression, and suppressed OPC differentiation; PAD4 inhibition partially reversed these abnormalities [90]. These findings link an extracellular inflammatory signal to an OPC-intrinsic epigenetic block on lineage progression in a defined vascular-risk context. However, the necessary in vivo source of TNF-α was not resolved, and the pathway should not be assumed to operate across all ischemic lesions.

Peripheral T cells can alter OPC fate after stroke primarily by reshaping the inflammatory niche, although direct T-cell–OPC interactions remain incompletely established. Experimental stroke studies identified infiltrating γδ T cells as an important source of IL-17 A during delayed injury [91], while IL-17 A and TNF-α can act cooperatively on astrocytes to increase CXCL1 production and neutrophil recruitment [92]. In inflammatory demyelination, IFN-γ can also induce antigen-processing and major histocompatibility complex programs in OPCs and increase their susceptibility to cytotoxic T-cell recognition [29]. These findings establish the capacity of T-cell-associated signals to alter antigenic state of OPCs, but they do not demonstrate that direct T-cell-mediated OPC injury is a major mechanism in adult ischemic stroke. Similarly, the IL-17 A/Notch1-associated oligodendroglial benefits reported with low-intensity pulsed ultrasound after stroke do not identify a specific peripheral T-cell population as the necessary source of IL-17 A [10]. Taken together, current evidence supports peripheral T-cell regulation of the environment in which OPCs survive and enter repair, but does not establish a necessary direct T-cell–OPC pathway governing lineage progression after adult ischemic stroke.

During later recovery, reparative myeloid programs may support white-matter repair by relieving successive niche and lineage bottlenecks. CD11c-positive microglia accumulate during post-stroke recovery and acquire phagocytic, lipid-handling, and myelin-supportive programs; disruption of this population impaired oligodendroglial-lineage recovery and white-matter restoration [93]. These findings support a role in establishing a permissive repair niche but do not identify a single OPC-directed effector. Within this context, exercise-induced microglial TREM2–activin A signaling provides more direct evidence that modification of the immune niche can promote OPC differentiation and remyelination through activin receptor signaling [9]. Peripheral regulatory T cells (Treg) can reinforce this reparative program. Treg-derived SPP1 acts through microglial integrins to enhance reparative microglial activity and subsequently oligodendrogenesis, while Treg depletion or microglial depletion compromises the corresponding white-matter benefit [94]. This evidence supports an indirect Treg–microglia–oligodendroglial route rather than a demonstrated direct Treg–OPC interaction. Microglial TNFR2 further links myelin-debris clearance and oligodendrocyte maturation, although this effect is sex-dependent [95]. The persistence of a permissive myeloid niche may also influence chronic repair. ZFP384 suppresses YY1-dependent chromatin interactions associated with recovery, whereas *Zfp384*-targeting antisense oligonucleotides prolong a reparative microglial program and improve chronic stroke recovery [96]. OPCs were not established as the principal downstream target; this finding is therefore best interpreted as evidence that sustained reparative myeloid states can condition later lineage progression, rather than as evidence of a direct ZFP384-dependent OPC mechanism. Collectively, these studies support a staged model in which niche clearance permits OPC differentiation, oligodendrocyte maturation, and eventual remyelination, but they do not establish a single linear immune pathway or show that every reparative myeloid state acts directly on OPCs.

Microglia-derived extracellular vesicles (EVs) provide a vesicle-mediated route through which reparative myeloid states can influence differentiation and maturation of surviving OPCs. In GPR17-lineage-traced mice subjected to permanent MCAO, regenerative microglia-derived EVs enhanced maturation of GPR17-lineage cells at lesion borders and improved neurological outcome. In vitro experiments implicated EV-associated transmembrane TNF in the pro-differentiating effect on OPCs [97]. A separate mouse ischemia study found that EVs from IL-4-conditioned microglia promoted oligodendrogenesis and white-matter repair; miR-23a-5p depletion attenuated these benefits, while reporter experiments identified *Olig3* as a direct miRNA target [98]. Together, these studies link defined microglial EV cargoes to differentiation- and maturation-associated oligodendroglial outcomes. However, because the EVs were administered experimentally, they do not establish that endogenous microglial EV release is necessary for post-stroke remyelination. The transmembrane-TNF finding also illustrates why TNF signaling should not be interpreted uniformly: its effects depend on ligand form, cellular source, receptor context, and the lineage state of the recipient.

Source-resolved stroke studies indicate that infiltrating MDMs can support the transition from early tissue injury toward later oligodendroglial repair, primarily through debris clearance and lesion-niche remodeling rather than through an established direct MDM–OPC signal. Microglia-specific *Hdac3* deletion increased acute chemokine-associated recruitment of CCR2-dependent macrophages to the infarct core, where myelin debris accumulated. Macrophage depletion and reconstitution experiments showed that the recruited cells accelerated debris clearance and contributed to subsequent oligodendrogenesis, white-matter integrity, and functional recovery [99]. In a separate study, early PLX5622-mediated microglial attenuation increased the recruitment of a GPNMB/CD63-enriched repair-associated macrophage population with lipid-metabolic and pro-angiogenic features. Transplantation of these macrophages improved white-matter integrity and vascular repair after transient MCAO [100]. Together, these studies indicate that resident microglia and MDMs can cooperate through recruitment and niche remodeling without performing interchangeable functions. However, these findings are intervention specific: enhanced repair after partial microglial attenuation does not imply that microglial depletion is generally beneficial. Indeed, broader microglia/macrophage *Sik3* deletion altered myelin phagocytosis and improved long-term white-matter integrity, but the manipulation did not resolve the contributions of the two myeloid origins [101]. Monocyte-specific *Ccr2* deficiency also impaired angiogenesis and functional recovery after permanent ischemia, supporting a reparative contribution of recruited monocytes but not a direct effect on OPC fate [102].

These source-resolved stroke studies support MDM contributions to oligodendroglial repair through debris clearance and niche remodeling, but do not establish that a direct MDM–OPC ligand–receptor interaction is necessary for endogenous repair in vivo. Candidate direct immune-to-OPC communication routes have instead been defined more clearly in non-ischemic demyelination models. Repair-associated macrophage-derived EVs enriched in cholesterol promote OPC differentiation and remyelination, with cholesterol manipulation and membrane-fusion experiments supporting a direct contribution of EV-associated lipid delivery [103]. Earlier work also implicated activin A released by repair-associated microglia/macrophages in OPC differentiation and remyelination [104]. Peripheral Tregs can likewise promote OPC differentiation through CCN3 in experimental demyelination, providing a direct mechanism distinct from the Treg–SPP1–microglia pathway established after stroke [105]. These studies strengthen the biological plausibility of direct immune-to-OPC signaling, but neither the operation nor the necessary cellular source of these pathways has been established in endogenous adult ischemic-stroke repair.

Host context further modifies the reparative capacity of myeloid cells: in aged stroke mice, impaired angiogenic and oligodendrogenic responses were partially restored by transplantation of young, but not aged, brain-derived microglia/macrophages [106]. This finding links myeloid-cell age to the capacity of the post-ischemic niche to support repair, but it does not identify OPCs as the sole or principal downstream target. Overall, stroke evidence is currently strongest for immune regulation of OPC survival, recruitment, and lineage progression through niche remodeling, whereas direct endogenous immune-to-OPC mechanisms remain supported mainly by cross-model evidence. Future studies should distinguish resident and infiltrating cell sources, test paired ligand–receptor perturbations, and determine whether immune-dependent changes in OPC survival or recruitment lead to differentiation, mature oligodendrocyte formation, newly formed myelin, and functional repair.

#### Astrocytic and inflammasome-associated signals shape the inflammatory niche encountered by OPCs

Astrocyte-derived inflammatory signals can compromise OPC survival and thereby reduce the progenitor pool available for subsequent recruitment and lineage progression. In a neonatal rat hypoxic–ischemic brain-injury model, short-chain fatty acid treatment reduced astrocytic NLRP3, IL-6, CCL2, and CXCL10 expression and was accompanied by reduced OPC loss. Complementary culture experiments implicated astrocytic SGK1–IL-6 signaling in this response [107]. Together, these findings link the astrocytic inflammatory state to OPC vulnerability and identify SGK1–IL-6 as a candidate astrocyte-to-OPC signaling route. However, the intervention altered multiple inflammatory mediators and did not establish the in vivo necessity of astrocyte-derived IL-6 or a defined receptor-mediated mechanism in OPCs. Moreover, the neonatal injury model differs substantially from adult focal ischemic stroke in developmental stage, immune composition, and OPC abundance, and the pathway therefore requires direct validation in adult stroke models. Inflammasome-associated signaling may amplify the post-ischemic inflammatory environment encountered by OPCs, but evidence for OPC-intrinsic inflammasome activation remains limited. An embolic-stroke study implicated TXNIP–NLRP3 signaling in inflammatory brain injury [108] (see the published erratum correcting duplicated representative panels [109]), whereas microglial STING–NLRP3 activation has been shown to promote GSDMD-associated pyroptosis [86]. These findings support the niche-level relevance of inflammasome-associated inflammation, but neither study establishes a direct inflammasome-dependent effect on OPC fate or a complete OPC-intrinsic TXNIP–NLRP3 cascade. Mature-oligodendrocyte pyroptosis is considered separately in Section "Pyroptosis and inflammatory propagation of oligodendrocyte loss" and should not be extrapolated directly to OPCs.

#### Integration of inflammatory and vascular signals under ischemic stress

Vascular and immune signaling should be interpreted as interacting components of the post-ischemic niche rather than as independent regulators of OPC fate. As detailed in Section "Ischemia reshapes OPC–vascular signaling", hypoxic OPC-derived VEGFA has been associated with barrier disruption and endothelial HSP90α has been linked to differentiation arrest, whereas appropriately timed HIF–Wnt signaling can support angiogenic OPC states. The direction of this response depends on oxygen severity, disease phase, vascular maturity, and whether the evidence derives from endogenous or transplanted OPCs [41, 42, 69, 70]. The immune compartment adds a further level of regulation: recruited monocytes and macrophages can participate in vascular remodeling, while changes in resident and infiltrating myeloid populations may alter both the inflammatory niche and the availability of signals supporting oligodendroglial repair [100, 102]. These findings support indirect myeloid regulation of the vascular–oligodendroglial niche but do not establish a necessary direct monocyte/macrophage-to-OPC signal. Together, these findings indicate that neuroimmune–oligovascular signaling is strongly context dependent rather than uniformly protective or detrimental. Enhanced angiogenesis or increased perivascular OPC abundance therefore does not establish BBB restoration or successful remyelination. The relevant outcome is whether vascular and immune remodeling creates a permissive environment for subsequent OPC lineage completion.

Taken together, post-ischemic immune and vascular signals regulate OPC fate in a sender-, phase-, and context-dependent manner rather than as uniformly protective or detrimental influences. Current stroke evidence is strongest for niche-mediated effects on OPC survival, recruitment, and differentiation, whereas direct endogenous ligand–receptor control of OPC fate and causal progression to newly formed myelin remain less established. Mechanistic and therapeutic claims should therefore specify the signal-producing cell, the affected lineage transition, whether the effect is direct or niche mediated, and the highest repair endpoint actually demonstrated.

## Spatiotemporal OPC state transitions and repair outcomes after ischemic stroke

The communication mechanisms outlined in Section "Convergent ischemic signaling across sequential OPC fate transitions" identify signals that can promote or constrain successive OPC transitions, but these effects do not by themselves demonstrate successful endogenous repair. Their significance must therefore be evaluated against the spatiotemporal distribution of lineage states and the progression from OPC survival and recruitment to mature oligodendrocyte generation and myelin restoration. The central issue is therefore not whether OPC numbers simply increase or decrease, but which OPC states occupy the infarct core, peri-infarct tissue, and remote white matter at each stage of recovery and whether those cells progress toward functional oligodendrocyte replacement. Severe injury can deplete the progenitor pool, whereas surviving cells may proliferate, redistribute, and enter reactive or differentiation-committed states; only a subset ultimately completes differentiation and remyelination. The balance among depletion, recruitment, arrest, and lineage completion changes substantially from the acute to chronic phase (Table 3).

**Table 3:** Spatiotemporal organization of OPC and oligodendroglial responses after ischemic stroke

Operational window or context	Dominant compartment	Representative OPC/lineage response	Potential consequence	Key evidence	Main limitation
Acute (hours–3 days)	Infarct core versus peri-infarct tissue	Core depletion; peri-infarct OPC increase by 48 h	Loss of repair pool or priming for recruitment	[7, 89, 112, 115]	Human acute data are extremely small; relative abundance is not lineage flux
Early subacute (3–14 days)	Peri-infarct cortex/striatum, SVZ, connected white matter, perivascular niche	Proliferation, migration, GPR17-lineage recruitment, angiogenic state selection; basal-ganglia OPCs + 139% and OLs + 126% at day 14	Tissue repopulation and vascular remodeling, but possible maturation delay	[43, 69, 110, 116, 117]	Different progenitor sources and marker-defined subsets are not interchangeable
Late subacute (7–30 days)	Damaged and remote white matter	Microglia-supported debris clearance and OPC differentiation; astrocytic or matrix-mediated arrest	Partial remyelination or persistent arrest; near-restoration of peri-infarct myelin density by 2 weeks in rat tMCAO	[9, 93, 95, 112, 121]	Strong dependence on model, sex, region, and inflammatory state
Chronic/later stage (≥ 4 weeks in most experimental studies; study-defined in human tissue)	Peri-infarct and connected tracts	Incomplete GPR17-lineage maturation at 8 weeks; human peri-infarct accumulation in later lesions (*n* = 34)	Neuronal preservation and network recovery, or chronic demyelination	[3, 8, 110, 111, 119]	Rodent remyelination exceeds demonstrated adult-human lineage turnover
Cross-phase host modifiers	Whole oligovascular and immune niche	Reduced proliferation, differentiation, or survival under aging, diabetes, and hyperhomocysteinemia; aged hypertensive rats show reduced callosal OPC proliferation at day 14	Reduced repair efficiency through impaired OPC survival, proliferation, or differentiation	[90, 123, 124]	Each modifier was tested in a specific model and cannot be generalized to all patients

This distinction is essential when interpreting post-stroke increases in PDGFRα-, NG2-, OLIG2-, GPR17-, or BCAS1-positive cells, because these markers span different oligodendroglial states and are not uniformly OPC specific [13, 110, 111]. Increased lineage-cell abundance therefore does not by itself demonstrate successful remyelination. Likewise, benefits observed with cultured or transplanted OPCs should not be attributed automatically to endogenous OPCs. Spatiotemporal analysis must consequently distinguish cell accumulation from lineage completion and endogenous responses from transplantation-based proof of principle.

**Fig. 5:**
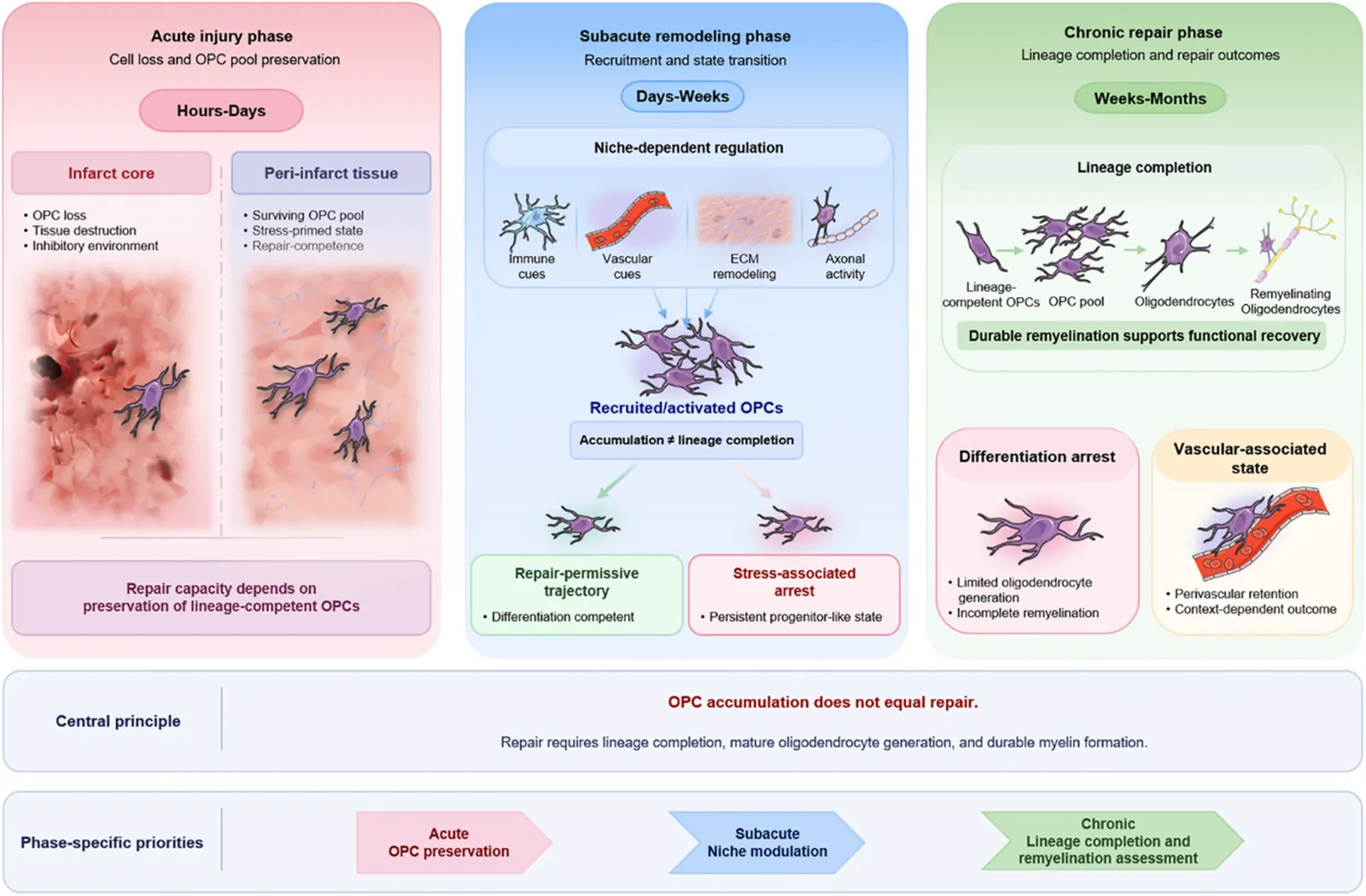
Conceptual framework of OPC fate transitions and repair trajectories after ischemic stroke. The framework integrates endogenous oligodendrocyte precursor cell (OPC) responses across lesion compartments and disease phases. (1) Acute phase: severe ischemia causes OPC loss and disrupts the cellular substrate available for later repair within the infarct core. In peri-infarct tissue, partial OPC survival permits reactive-state priming, with early responses shaped by spatial context. (2) Subacute phase: recruitment, proliferation, and migration vary among regions and do not necessarily indicate maturation. Immune, extracellular-matrix, vascular, and axonal signals form a context-dependent fate gate that biases OPCs toward oligodendrogenic, differentiation-arrested, or vascular-associated states. (3) Chronic phase: successful lineage completion can generate mature oligodendrocytes, but subsequent formation of stable new myelin and functional benefit remain distinct downstream endpoints. Alternatively, persistent immature OPC accumulation may reflect differentiation arrest, whereas vascular-associated states may represent supportive or maladaptive outcomes depending on context; neither outcome alone demonstrates remyelination. (4) Integrated interpretation: OPC abundance, lineage completion, mature oligodendrocyte generation, new myelin formation, and functional recovery represent distinct biological endpoints. The phase-specific priorities shown are acute OPC preservation, subacute niche modulation, and chronic assessment of lineage completion and remyelination. The phases overlap, lesion compartments coexist, and host context can modify each transition. Because human tissue provides state snapshots rather than longitudinal lineage histories, this framework represents a testable synthesis rather than an established sequence in humans [3, 8, 69, 89, 93, 110–112, 116–121]

For the purposes of this review, acute, early subacute, late subacute, and chronic phases are used as overlapping operational windows rather than fixed biological boundaries (Table 3). Fig. 5 organizes these windows along a conceptual fate trajectory but does not imply that every lesion compartment or OPC population passes through them synchronously or in a fixed sequence. The timing and cellular composition of each window vary with reperfusion, lesion severity, anatomical compartment, and host context. Accordingly, findings from infarct core, peri-infarct gray matter, and connected white-matter tracts should not be pooled into a single recovery trajectory.

### Acute OPC depletion and early state priming

Acute OPC responses are spatially divergent, with lesion compartment determining the size of the viable progenitor pool available for later repair. In adult rats subjected to transient middle cerebral artery occlusion, the infarct core showed progressive loss of OPCs, mature oligodendrocytes, and myelin beginning by 48 h, whereas the peri-infarct region showed milder oligodendrocyte and myelin loss together with a small early increase in OPCs [112]. Thus, acute core depletion can coexist with peri-infarct OPC retention or accumulation. Whole-lesion averages can obscure these opposing trajectories, and an early increase in peri-infarct OPC number should not be interpreted as evidence of proliferation, differentiation competence, or eventual lineage completion.

Early OPC loss can constrain the size of the progenitor pool available for later oligodendrogenesis. Intervention studies support this principle but differ in lineage specificity and causal strength. WIN55,212-2 administered after permanent MCAO reduced injured Tau1-positive/NG2-positive cells and increased proliferation of NG2-positive cells, although NG2 is not lineage exclusive [113]. Necrostatin-1 also preserved OPCs and white matter after transient MCAO, but its prophylactic design and non-lineage-specific pathway measurements limit mechanistic attribution [77]. More lineage-focused evidence showed that ferrostatin-1 treatment and *Plin2* knockdown reduced peri-infarct OPC loss, subsequent myelin injury, and neurological deficits [7]. However, the accompanying myelin and functional improvements do not by themselves demonstrate that the preserved OPCs subsequently differentiated and generated new myelin. Taken together, these studies support acute OPC preservation as a means of retaining the cellular substrate for later repair, but pool preservation remains an earlier endpoint than lineage completion or remyelination.

Surviving OPCs also undergo rapid state changes rather than simply maintaining a homeostatic phenotype. Single-nucleus sequencing in rodent stroke identified infarct-restricted proliferating OPCs with CD44-associated reactive features and transcriptional overlap with reactive astrocytes, while osteopontin-positive myeloid cells localized near these cells and osteopontin enhanced OPC migration in vitro [89]. Spatial and single-cell profiling across the first post-ischemic week further identified inflammatory-responsive oligodendroglial states distributed differently across the lesion core, peripheral scar, and surrounding tissue [114]. Together, these findings support spatially structured early OPC state priming, but transcriptomic state labels, spatial proximity, and inferred cell–cell interactions do not establish fixed OPC subtypes or necessary in vivo signaling pathways. They also do not determine whether the identified states represent transient stress responses, obligatory intermediates in repair, or progenitors that subsequently complete differentiation and generate new myelin.

Acute human evidence is limited but consistent with early oligodendroglial disruption. Single-nucleus RNA sequencing of peri-infarct cortex resected within approximately 24 h identified relatively more OPCs, fewer mature oligodendrocytes, and trajectories shifted toward earlier oligodendroglial states [115]. However, the dataset comprised only two stroke specimens and three trauma-derived comparators. Relative OPC enrichment could reflect differential survival, sampling location, altered nuclear recovery, or recruitment and therefore does not establish proliferation or reparative lineage progression. These findings should be interpreted as a cross-sectional snapshot of the acute human oligodendroglial state rather than as evidence of a longitudinal OPC fate trajectory, and they require validation in larger, spatially resolved cohorts.

### Subacute recruitment and divergence of reactive OPC states

#### Recruitment is region-, source-, and lineage-state dependent

Subacute changes in OPC abundance are strongly region dependent, but cell counts alone cannot determine whether the observed accumulation reflects local proliferation, differential survival, or migration. In rat transient MCAO, peri-infarct basal ganglia showed concurrent increases of 139% in PDGFRα-positive OPCs and 126% in CC1-positive oligodendrocytes at day 14 relative to day 1. In contrast, cortical OPCs increased without a corresponding significant increase in mature oligodendrocytes, whereas both populations remained low in the peri-infarct corpus callosum after an initial decline. Oligodendroglial abundance also correlated only partly with local vessel density [116]. These regional differences indicate that post-stroke OPC expansion does not constitute a uniform hemispheric oligodendrogenic response and that OPC accumulation and lineage progression can become spatially uncoupled.

Lineage-tracing studies further indicate that the subacute reactive pool has heterogeneous origins and fates. Ascl1-expressing progenitors labeled during days 2–6 expanded in the ipsilateral subventricular zone, striatum, and corpus callosum and generated mature oligodendrocytes by days 30–60, although Ascl1 marks both neural and oligodendroglial progenitors and therefore does not specifically trace resident parenchymal OPCs [117]. GPR17-lineage tracing provides a more oligodendroglial-lineage-focused view: GPR17-expressing OPCs accumulated around the lesion from day 3 to 2 weeks through proliferation and recruitment, yet most BrdU-labeled cells retained NG2 and only a subset acquired mature oligodendrocyte markers by 8 weeks [110]. These studies support two distinct conclusions. Ascl1 tracing demonstrates that a mixed post-stroke progenitor pool can generate mature oligodendrocytes, whereas GPR17 tracing shows that expansion of a more lineage-focused population is frequently followed by incomplete maturation. Neither study quantifies the contribution of the labeled progenitor source to newly formed myelin.

#### The subacute niche selects divergent reparative states

The subacute niche can determine whether expanded or recruited OPCs progress toward maturation or remain arrested. Pericyte deficiency impaired OPC differentiation and remyelination despite preserved peri-infarct OPC proliferation, demonstrating that OPC expansion alone is insufficient for lineage completion [118]. The immune evidence detailed in Section "Resident and peripheral immune cells regulate OPC injury and repair" places microglial programs at distinct checkpoints in this transition. Disruption of the CD11c-positive microglial population impaired oligodendroglial-lineage recovery and white-matter restoration [93]. Exercise-induced TREM2–activin A signaling provided more direct evidence for microglia-to-OPC regulation of differentiation and remyelination [9], while conditional microglial deletion of *Tnfrsf1b* (encoding TNFR2) altered myelin-debris clearance and oligodendrocyte maturation in a strongly sex-dependent manner [95]. These interventions do not define a single linear pathway. Instead, they place the subacute bottleneck at the level of niche permissiveness, including the removal of inhibitory debris and the availability of signals compatible with OPC differentiation and oligodendrocyte maturation.

The contribution of infiltrating myeloid cells adds a further spatial dimension to this response. Source-resolved studies indicate that recruited macrophages can concentrate within the infarct core and facilitate myelin-debris clearance, whereas repair-associated MDM populations induced by early microglial attenuation can support subsequent white-matter and vascular remodeling [99, 100]. The highest demonstrated outcomes in these studies are therefore debris clearance and tissue-niche remodeling rather than a direct MDM-to-OPC fate signal. They do not establish that macrophage activity within the infarct core directly causes OPC differentiation or lineage completion in peri-infarct tissue. The distinction between core-associated debris clearance and peri-infarct lineage completion therefore remains important when interpreting whole-lesion measures of oligodendroglial recovery.

Subacute hypoxia may bias surviving OPCs toward vascular-associated programs without establishing irreversible loss of oligodendroglial potential. After transient MCAO, perivascular OPC accumulation accompanied angiogenesis, and hypoxic OPCs acquired a pro-angiogenic secretory profile; delayed administration of OPC-conditioned medium enhanced vascular growth and functional recovery [43]. An integrated stroke atlas subsequently proposed that severe hypoxia favors angiogenic OPC states during the subacute phase, whereas milder hypoxia is associated with more oligodendrogenic states during later recovery [69]. This framework offers a plausible explanation for why OPCs may accumulate near vessels without promptly generating myelin. However, it integrates transcriptomic inference with conditioned-medium and transplantation-based evidence and therefore should be regarded as a working model of state divergence rather than a validated endogenous OPC fate switch. The available evidence does not track individual endogenous OPCs through this transition or determine whether vascular-associated states preclude subsequent oligodendrocyte differentiation.

### Chronic remyelination versus differentiation arrest

#### Endogenous remyelination occurs but is frequently incomplete

Evidence that endogenous oligodendroglial progression can reach mature-cell and remyelination-associated endpoints after ischemic stroke comes from convergent but non-equivalent observations. In rat transient MCAO, peri-infarct OPC expansion was followed by recovery of mature oligodendrocyte number and near-restoration of myelin density by 2 weeks, although lineage tracing was not available to establish a precursor–product relationship [112]. Ascl1-lineage tracing subsequently demonstrated generation of mature oligodendrocytes in ischemic striatum and corpus callosum at 30–60 days [117], while permanent ischemia was associated with early OPC recruitment, emergence of 3R-Tau-positive premyelinating cells, and long-term remyelination [119]. Together, these studies establish the biological feasibility of post-stroke oligodendrogenesis and remyelination. However, no single study combines OPC-specific lineage tracing with direct measurement of newly formed compact myelin, regional repair efficiency, and functional outcome. The extent and sufficiency of endogenous repair therefore cannot be generalized across lesion compartments, models, ages, or human stroke.

Convergent evidence places an important chronic-repair bottleneck between OPC accumulation and successful maturation. In rats, OPC abundance recovered between days 7 and 28 and OLIG1 returned to the nucleus, whereas myelin basic protein recovered only partially and remained below control levels [120]. Because myelin basic protein is a tissue-level readout, its recovery does not identify the cellular origin or demonstrate newly generated myelin. GPR17-lineage tracing similarly showed that most recruited cells remained undifferentiated even at 8 weeks [110]. In a focal white-matter stroke model, reactive astrocyte-derived inhibin A suppressed OPC Matrilin-2 expression and differentiation during a limited-regeneration phase, whereas restoration of Matrilin-2 enhanced remyelination and motor recovery [121]. Together, these studies show that numerical recovery of the OPC pool can coexist with persistent differentiation arrest and incomplete myelin restoration. Chronic repair failure can therefore reflect niche-dependent restriction of lineage progression even when the acute progenitor pool has been partially replenished.

#### Human lesions reveal oligodendroglial accumulation without proportional maturation

Human stroke tissue provides convergent cross-sectional evidence of oligodendroglial-lineage accumulation without a proportional increase in markers of later maturation. In six post-mortem stroke cases, peri-infarct cortex and white matter contained increased OLIG2-positive oligodendroglial-lineage cells and early-stage BCAS1-positive cells without a corresponding increase in late-stage BCAS1-positive cells, consistent with relative enrichment of earlier lineage states but not direct evidence that individual cells became differentiation arrested [111] (with a linked corrigendum [122]). A larger lesion-stage analysis similarly identified GPR17-positive/BCAS1-positive differentiation-committed OPCs accumulating in later peri-infarct lesions, with their distribution associated with reactive microglia/macrophages and astrocytic scarring and showing sex-dependent relationships in chronic tissue [8]. These spatial associations do not establish that the neighboring glial responses caused the observed oligodendroglial states. Because both studies were observational and lesion age was heterogeneous, they cannot measure lineage flux directly or determine how much newly formed myelin arose from the accumulated cells. Nevertheless, they support the interpretation that OPC accumulation in human stroke lesions is not equivalent to successful remyelination.

#### Remyelination has causal consequences for neuronal survival

Opposing OPC-specific genetic perturbations provide the strongest causal evidence that completion of oligodendroglial lineage progression contributes to neuronal preservation after stroke. After mouse transient MCAO, oligodendrocyte loss and demyelination persisted into the subacute and chronic phases, while newly generated myelin remained incomplete. Conditional *Olig2* deletion in OPCs further impaired remyelination, neuronal survival, and functional recovery, whereas conditional deletion of *Chrm1* (encoding the inhibitory muscarinic M1 receptor) in OPCs enhanced remyelination and improved neuronal and white-matter preservation [3]. Clemastine treatment produced similar benefits, although its effects are less lineage specific because the drug can act on multiple cell populations. Because impairing OPC lineage progression worsened repair whereas releasing an OPC-intrinsic differentiation constraint improved it, these findings extend beyond associations based on cell number or lineage markers and support a causal contribution of lineage completion and myelin restoration to neuronal and functional recovery. They do not, however, establish that every behavioral benefit is mediated exclusively by newly formed myelin.

#### Host factors and continuing white-matter injury constrain lineage completion

Host factors modify different bottlenecks along the OPC repair trajectory rather than imposing a single uniform constraint on lineage completion. In hypertensive rats, older animals showed fewer early PDGFRα-positive OPCs and persistently reduced proliferation after focal ischemia despite later recovery of cell number [123], indicating that numerical restoration does not necessarily reflect recovery of progenitor competence. Diabetes impaired OPC proliferation, generation of new myelinating oligodendrocytes, white-matter conduction, and reparative microglial responses after distal MCAO; high-glucose cocultures further showed microglia-associated suppression of OPC differentiation [124]. Hyperhomocysteinemia imposed a distinct differentiation block through TNF-α/PAD4-associated histone citrullination and reduced myelin regulatory factor (*Myrf*) expression, which was partially reversed by PAD4 inhibition [90]. These findings place age and hypertension primarily at the level of progenitor availability and proliferative competence, diabetes across both OPC-intrinsic and niche-supported repair processes, and hyper-homocysteinemia at a defined differentiation checkpoint. These model-specific effects should not be treated as interchangeable or generalized to all ischemic lesions.

Net myelin recovery depends on the balance between the generation of new oligodendrocytes and the continuing loss of pre-existing mature oligodendrocytes in connected white matter. Astrocyte-derived LCN2 has been linked to both LRP2/JNK3-associated oligodendrocyte degeneration and caspase-1/GSDMD-associated pyroptotic injury in the contralateral corpus callosum after stroke [78, 81]. These studies identify ongoing mature-oligodendrocyte injury rather than an OPC-intrinsic fate mechanism. Consequently, an active or numerically restored progenitor response can coexist with persistent net myelin loss when degeneration of existing oligodendrocytes offsets the contribution of newly generated cells.

### Functional consequences beyond oligodendrocyte replacement

#### Vascular remodeling is beneficial or maladaptive according to context

Evidence for non-myelinating OPC functions after stroke is most developed for vascular remodeling, although the supporting studies operate at different levels of causal inference. Perivascular OPC accumulation and hypoxia-induced pro-angiogenic secretion after transient MCAO support the capacity of OPCs to influence the post-ischemic vasculature [43], while transcriptomic analyses have proposed a subacute angiogenic OPC state under more severe hypoxic conditions [69]. Pharmacological stabilization of OPC HIF-1α through PHD2 inhibition further increased WNT7A-dependent endothelial angiogenesis [70]. Conditioned-medium experiments demonstrate pro-angiogenic secretory capacity, transcriptomic analyses identify a candidate endogenous state, and pathway stabilization demonstrates experimental inducibility. Together, these findings support the angiogenic potential of OPCs but do not establish that endogenous OPC-derived signaling is necessary for post-stroke vascular repair or that the magnitude of experimentally induced responses lies within the physiological range.

Perivascular OPC accumulation is not inherently reparative. Under chronic cerebral hypoperfusion, OPC-derived VEGFA was associated with endothelial junction disruption and early BBB leakage, while circulating VEGFA correlated with vascular permeability in human small-vessel disease but could not be assigned specifically to OPCs [41]. Endothelial caveolin-1 loss and HSP90α secretion also promoted abnormal vessel-associated OPC retention and impaired oligodendrogenesis during chronic ischemia [42]. These findings show that vascular proximity and angiogenic signaling can accompany barrier dysfunction or lineage arrest, depending on endothelial state and ischemic context. However, because the evidence derives mainly from chronic hypoperfusion and small-vessel-disease settings, the same mechanisms cannot be assumed to operate unchanged after adult focal ischemic stroke. Perivascular OPC abundance should therefore be treated as a spatial or state-associated readout rather than as evidence of BBB restoration, oligodendrocyte generation, or successful remyelination.

OPC transplantation provides a therapeutic proof of principle that should be distinguished from evidence of the endogenous OPC fate trajectory. Exogenous OPCs have reduced BBB leakage, enhanced angiogenesis and remyelination, and promoted neurite growth and synaptogenesis after MCAO, with Wnt/β-catenin-associated signaling implicated in some of these effects [11, 44, 45]. More recent transplantation studies also reported reduced inflammatory cytokines, neutrophil infiltration, and BBB disruption [125]. These broad outcomes demonstrate the therapeutic capacity of experimentally delivered OPCs, but they do not determine whether each benefit requires graft differentiation and myelin formation or instead reflects paracrine, vascular, immune, or neurotrophic effects. Transplanted cell dose, source, state, and spatial distribution also differ from those of the endogenous surviving OPC pool. Transplantation evidence should therefore not be mapped directly onto the endogenous trajectory summarized in Fig. 5 or used to infer that resident OPCs exert effects of comparable magnitude or direction.

#### Evidence is asymmetric between immune-to-OPC and OPC-to-immune signaling

As detailed in Section "Resident and peripheral immune cells regulate OPC injury and repair", stroke studies provide stronger evidence for immune-to-OPC regulation, acting either directly or through debris clearance and niche remodeling, than for endogenous OPC-to-immune signaling [9, 93–95]. By contrast, evidence that resident OPCs broadly suppress or amplify post-stroke inflammation derives mainly from transcriptomic inference, culture systems, or transplantation studies. Reduced inflammation after OPC transplantation is therapeutically relevant but does not establish a constitutive immunoregulatory function of endogenous OPCs. The current evidence therefore supports a directional asymmetry: immune cells can alter OPC survival and lineage progression, whereas a general endogenous OPC-to-immune regulatory function remains unproven. Establishing the reverse direction will require OPC-specific perturbation linked to immune-cell outcomes while distinguishing primary OPC-to-immune effects from secondary inflammatory changes caused by altered tissue injury or myelin damage.

#### Evidence for axonal and network recovery is strongest for completed lineage function

As discussed in Section "Remyelination has causal consequences for neuronal survival".  OPC-specific manipulation of myelinogenesis provides the strongest causal OPC-related evidence linking lineage completion and myelin restoration to neuronal preservation and functional recovery [3]. Transplantation studies additionally report enhanced neurite growth and synaptogenesis, but these effects cannot be attributed automatically to equivalent functions of resident OPCs [11]. Accordingly, current evidence supports a myelin-mediated contribution of the oligodendroglial lineage to neuronal and network recovery more strongly than a direct endogenous OPC-to-neuron trophic or synaptic mechanism. Establishing such a direct mechanism will require OPC-specific perturbation of candidate signals while separating neuronal effects from the secondary consequences of altered myelin repair.

### An integrated spatiotemporal model and human constraints

OPC responses after ischemic stroke can be conceptualized across four interacting dimensions: lesion compartment, time, lineage state, and host context. Severe core ischemia favors progenitor loss, whereas partially perfused peri-infarct tissue permits survival, proliferation, and migration. During the subacute phase, vascular, inflammatory, and extracellular-matrix cues can shift surviving OPCs toward reactive, angiogenic, differentiation-competent, or arrested states. Chronic recovery then depends on whether surviving and recruited OPCs progress through lineage commitment, escape inhibitory niche signals, and generate stable myelin. Age, metabolic and vascular comorbidities, sex, and other host factors modify the probability of each transition. This framework explains why OPC expansion can coexist with failed remyelination. Figure 5 summarizes these transitions from acute changes in progenitor-pool size and state priming through subacute recruitment and state divergence to chronic lineage completion or arrest, while distinguishing OPC accumulation from mature oligodendrocyte generation, new myelin formation, and functional recovery.

Human evidence captures only fragments of this spatiotemporal trajectory. Acute surgical tissue suggests relative enrichment of early OPC states together with loss of mature oligodendrocytes, but the available single-nucleus dataset includes only two stroke cases [115]. Post-mortem studies identify early BCAS1-positive and GPR17-positive/BCAS1-positive oligodendroglial states in peri-infarct tissue without proportional maturation and show associations with neighboring glial responses [8, 111], while persistent myelin loss and denuded axons remain evident in chronic human lesions [3]. None of these datasets provides longitudinal lineage tracing or direct measurements of transition rates within the same individuals. Cross-sectional observations from different patients and lesion stages cannot reconstruct a within-patient lineage history, and the timing or efficiency observed in rodent models cannot be transferred directly to human stroke. The sequence summarized in Fig. 5 should therefore be regarded as a testable synthesis of current evidence rather than an established human natural history.

## Translating OPC biology into therapeutic strategies

Therapeutic targeting of OPCs should focus on the specific lineage transition that limits repair rather than on increasing progenitor abundance alone. Table 4 therefore organizes candidate strategies according to the biological transition they target rather than by drug class alone.

**Table 4:** Transition-based therapeutic strategies for OPC-centered repair after ischemic stroke

Therapeutic transition	Representative approach	Highest demonstrated endpoint	Key evidence	Major translational limitation
Preserve the acute repair pool	Ferrostatin-1/PLIN2 targeting	Reduced OPC loss, later myelin injury, and deficits	[7]	Systemic and non-lineage-specific intervention
Combine early protection with later maturation	Low-intensity pulsed ultrasound	New mature OLs, myelin, and functional recovery	[10]	Stimulation parameters and human target engagement unresolved
Release an intrinsic differentiation brake	OPC-specific *Chrm1* deletion; clemastine	New myelin, neuron survival, and functional recovery	[3]	Drug is less selective than the genetic proof
Promote lineage completion	Agomelatine; anti-LINGO-1; miR-219; FGF17	Enhanced oligodendrogenesis or remyelination with behavioral improvement	[127–130]	Preclinical and often pleiotropic
Remove niche-mediated arrest	Matrilin-2; cGAS–STING inhibition	Remyelination and functional recovery; delayed-onset efficacy demonstrated for cGAS–STING inhibition	[121, 133]	Model-specific and not OPC intrinsic
Restore myeloid debris metabolism	SLC11A1 inhibition; LAL/HPβCD	Debris clearance and remyelination after white-matter stroke	[136, 137]	Requires validation in aged territorial stroke
Enhance OPC–endothelial angiogenic signaling	Oridonin–PHD2–HIF-1α–WNT7A	Increased endothelial angiogenesis	[70]	Angiogenesis does not establish BBB repair or remyelination
Supply engineered cells or signals	OPC transplantation; hypoxia-conditioned OPC products; stem-cell-derived exosomes	BBB, vascular, myelin, neuronal, and behavioral effects	[11, 44, 45, 69, 131]	Product heterogeneity, delivery, safety, and uncertain donor-versus-paracrine mechanisms

Interpretation of therapeutic efficacy also requires an evidence hierarchy. OPC-specific conditional manipulation linked to new myelin formation and functional outcome provides stronger causal support than changes in NG2 or PDGFRα immunoreactivity alone. Lineage-resolved mature oligodendrocyte measurements and ultrastructural evidence of myelin restoration are more informative than total myelin-protein abundance, which may reflect preservation of pre-existing myelin. Interventions acting through microglia, astrocytes, endothelium, or transplanted cells may improve the OPC environment without establishing an OPC-intrinsic mechanism. OPC-directed strategies should therefore be considered experimental adjuncts to, rather than substitutes for, timely reperfusion and standard stroke care.

### A stage-matched therapeutic framework

Therapeutic timing should be matched to the dominant biological bottleneck rather than to calendar time alone. During the acute phase, limiting OPC and oligodendrocyte loss may preserve the cellular substrate for subsequent repair. In the early subacute phase, recruitment and proliferation are likely to be beneficial only when the vascular and inflammatory niche remains permissive for further lineage progression. During later recovery, the limiting steps may shift toward debris clearance, release from differentiation arrest, and stabilization of newly generated oligodendrocytes and myelin. Because these transitions vary with lesion severity, white- versus gray-matter involvement, age, and reperfusion status, treatment windows should ultimately be defined by biological state as well as time after stroke. These stage assignments represent a biological framework rather than clinically validated treatment windows. The timing of intervention should be interpreted in relation to the actual treatment onset and duration tested in each study, and efficacy after early initiation should not be assumed to establish efficacy when treatment is first introduced during chronic recovery.

Therapeutic target engagement should be distinguished from successful tissue repair. Reduced OPC death, increased proliferation, acquisition of mature oligodendrocyte markers, formation of new compact myelin, restoration of axonal conduction, and functional recovery represent successive but non-equivalent endpoints. Interventions that achieve only progenitor survival or expansion should therefore be described as OPC preservation or recruitment rather than remyelination. Conversely, behavioral improvement without lineage-resolved evidence may be therapeutically meaningful but cannot establish that OPC-mediated repair caused the benefit. This endpoint hierarchy provides a consistent framework for evaluating the therapeutic evidence discussed below.

### Preserving the acute OPC and oligodendroglial pool

Ferroptosis inhibition provides one of the more direct approaches to acute OPC preservation. In mouse cerebral ischemia/reperfusion, ferrostatin-1 reduced peri-infarct OPC loss and subsequent myelin injury, while *Plin2* knockdown attenuated demyelination and neurological deficits [7]. These findings support PLIN2-associated ferroptotic injury as a candidate therapeutic axis linking acute progenitor survival to later white-matter outcome. However, *Plin2* knockdown was not genetically restricted to OPCs, and systemic ferroptosis inhibition can protect multiple ischemia-vulnerable cell populations. Its therapeutic value as an OPC-directed strategy therefore requires evidence that lineage-specific preservation contributes benefit beyond general cytoprotection.

Low-intensity pulsed ultrasound provides a nonpharmacological strategy that spans both acute oligodendroglial preservation and later lineage progression. In male mice, daily stimulation reduced early OPC and oligodendrocyte loss, preserved white matter, and subsequently increased newly generated APC-positive oligodendrocytes and myelin without simply expanding the PDGFRα- or NG2-positive precursor pool [10]. These effects were associated with suppression of IL-17 A/Notch1 signaling. The combined preservation and maturation phenotype is therapeutically attractive, although the optimal stimulation parameters, brain penetration, sex generalizability, and causal requirement for IL-17 A/Notch1 signaling remain unresolved.

Edaravone dexborneol provides a useful clinical–preclinical bridge, although the human evidence is not OPC specific. In a nonrandomized study of 73 patients with first left basal-ganglia infarction, treatment was associated with improved diffusion-tensor and network measures at 3 months, while parallel MCAO and primary-OPC experiments linked the drug to enhanced OPC proliferation and differentiation through Akt/mTOR-associated signaling [126]. The clinical imaging findings increase translational relevance but do not establish that OPC engagement mediated the observed benefit. Treatment selection, baseline imbalance, and the pleiotropic antioxidant and anti-inflammatory actions of edaravone dexborneol remain potential confounders. This study therefore supports translational plausibility rather than human validation of an OPC-specific mechanism.

### Driving lineage completion and functional remyelination

The strongest current evidence for therapeutically enhancing lineage completion comes from OPC-specific manipulation of myelinogenesis. Conditional deletion of *Chrm1* (encoding the inhibitory muscarinic M1 receptor) in OPCs enhanced post-stroke remyelination, neuronal survival, white-matter integrity, and functional recovery, whereas OPC-specific *Olig2* deletion produced the opposite phenotype [3]. These reciprocal genetic manipulations provide strong causal evidence that successful OPC-derived myelin repair contributes to stroke recovery. Clemastine reproduced the promyelinating direction pharmacologically, with treatment initiated 1 day after tMCAO and continued for 8 weeks at 10 mg/kg/day in mice [3]. However, its effects are less lineage specific because it acts on multiple cell populations and carries anticholinergic and sedating liabilities. This regimen supports early-initiated, sustained treatment but does not establish the efficacy of delayed chronic-phase initiation. Dedicated stroke studies are therefore still needed to define its treatment window, dose, and safety in older patients.

Several pharmacological approaches further support oligodendroglial differentiation as a druggable checkpoint, although with lower mechanistic specificity than OPC-restricted genetic manipulation. Agomelatine promoted OPC differentiation and remyelination through an LRP1/PPARγ-associated mechanism in vitro, ex vivo, and after transient MCAO, with little effect on OPC proliferation [127]. Anti-LINGO-1 treatment after photothrombotic stroke increased myelinating APC-positive oligodendrocytes, corticospinal myelin, and functional recovery [128], while miR-219 agonism promoted oligodendrocyte maturation, improved myelin ultrastructure, and reduced cognitive impairment after MCAO/reperfusion, with LINGO-1 implicated as a downstream target [129]. These studies support differentiation as a therapeutically accessible checkpoint, but none yet combines OPC-specific target engagement, direct demonstration of newly formed myelin, replication across stroke models, and human validation.

Broader regenerative strategies may engage oligodendroglial repair while acting simultaneously on other cellular compartments. Intranasal FGF17 improved motor and cognitive recovery after photothrombotic stroke, promoted oligodendrogenesis through ERK–SRF-associated signaling, and also supported neuronal survival and axonal regeneration through additional pathways [130]. Dental-pulp-stem-cell-derived exosomes administered after MCAO promoted OPC differentiation and white-matter repair through an exosomal MYL9–PRMT5–ID2-associated mechanism [131]. These approaches may be advantageous when neuronal and oligodendroglial repair must be restored together, but their pleiotropic actions complicate attribution of functional benefit specifically to OPCs. For exosome-based therapy, cargo heterogeneity, manufacturing consistency, biodistribution, and invasive delivery remain substantial translational barriers.

To establish causal dependence on lineage completion, promyelinating studies should, where feasible, test whether selective blockade of OPC differentiation attenuates the observed remyelination and functional benefits.

### Reprogramming the inflammatory, metabolic, and extracellular niche

Extrinsic niche barriers may need to be relieved before direct stimulation of OPC differentiation can produce effective repair. In focal white-matter stroke, reactive astrocyte-derived inhibin A suppressed OPC Matrilin-2 expression and differentiation, whereas restoration of Matrilin-2 enhanced oligodendrocyte differentiation, ultrastructural remyelination, and motor recovery [121]. Human and mouse white-matter lesions also accumulated macrophage-derived type I collagen associated with enhanced gliosis and impaired oligodendrocyte differentiation and remyelination [132]. The Matrilin-2 study provides functional rescue evidence, whereas collagen accumulation currently represents a target-discovery observation rather than a validated therapeutic intervention. These findings identify extracellular-matrix composition as an active regulator of lineage completion rather than a passive feature of the post-stroke scar.

Myeloid debris processing represents a second niche-level therapeutic checkpoint. Genetic or pharmacological inhibition of cGAS–STING initiated as late as 3 days after transient MCAO altered phagocyte state, enhanced myelin-debris uptake and degradation, supported oligodendrocyte maturation and remyelination, and improved long-term recovery [133]. Selective inhibition of soluble TNF with XPro1595 likewise improved myelin integrity and functional outcome while modifying microglial, astrocytic, and oligodendroglial responses [134]. Neither intervention is OPC intrinsic; their therapeutic rationale is instead to create a permissive environment for lineage progression. Demonstrating that functional benefit depends on downstream OPC differentiation would strengthen causal attribution to oligodendroglial repair.

Rehabilitation can also remodel the post-stroke niche through multicellular immune mechanisms. Exercise initiated 48 h after transient MCAO increased microglial *TREM2* and activin A, promoted OPC differentiation and remyelination, and improved cognitive outcome; Trem2 silencing or activin A neutralization attenuated these effects [9]. A complementary study showed that exercise recruited regulatory T cells through CXCL12/CXCR4-associated signaling and enhanced osteopontin-dependent myelin-debris clearance and white-matter repair [135]. These pathways may represent complementary components of rehabilitation-induced niche remodeling rather than mutually exclusive mechanisms. Their translational application, however, should account for lesion severity, exercise dose, treatment onset, and patient feasibility.

Recent studies identify lysosomal iron and lipid handling in reparative microglia as tractable determinants of white-matter repair. After white-matter stroke, SLC11A1-dependent lysosomal iron accumulation impaired myelin-debris processing, whereas deferoxamine, microglial *Slc11a1* knockdown, and pharmacological SLC11A1 antagonism improved debris clearance and recovery [136]. In a related metabolic pathway, lysosomal acid lipase was required for GPNMB-positive microglia to hydrolyze cholesteryl esters and support remyelination, and hydroxypropyl-β-cyclodextrin restored remyelination in a poorly reparative white-matter-stroke setting [137]. These findings shift therapeutic attention from broad suppression of inflammation toward restoration of the metabolic capacity required for effective myelin-debris clearance. Their relevance to large territorial infarction, aged microglia, and human stroke remains to be established.

Recruitment and functional reprogramming of peripheral macrophages represent complementary, but less clinically mature, approaches to niche-directed repair. Experimental microglial *Hdac3* deletion and early microglial attenuation have demonstrated that altering resident–infiltrating myeloid interactions can improve debris clearance or generate macrophage populations with reparative properties [99, 100]. These findings do not support indiscriminate microglial depletion or systemic enhancement of monocyte recruitment. Their translational value will depend on identifying the responsible macrophage states, defining a safe treatment window, and determining whether the benefits persist in aged and comorbid animals. Immune-derived EVs provide another potential means of delivering defined pro-differentiating signals, but their cargo, cellular specificity, biodistribution, and contribution to endogenous versus treatment-induced repair require further validation [97, 98]. Mechanistic evidence from non-ischemic demyelination models further supports the potential of macrophage-derived EVs to promote OPC differentiation, although its relevance to ischemic stroke remains to be established [103].

Niche-directed therapy must also account for host context. Microglial *Tnfrsf1b* deletion impaired myelin-debris clearance, oligodendrocyte maturation, and recovery predominantly in female mice, with weaker or partly divergent effects in males [95], indicating that this pathway should not be assumed to be sex neutral. Hyperhomocysteinemia creates a different therapeutic context in which TNF-α/PAD4-associated histone citrullination suppresses myelin regulatory factor (*Myrf*) expression and OPC differentiation; PAD4 inhibition partially restored white-matter repair [90]. These findings support a biomarker-defined approach in which the value of a niche target depends on sex, inflammatory state, and vascular-risk background rather than on pathway activation alone.

### Oligovascular and multimodal interventions

OPC-associated angiogenic signaling represents a potential therapeutic component of oligovascular repair. Hypoxic OPCs acquire a pro-angiogenic secretory profile, and delayed administration of OPC-conditioned medium after transient MCAO enhanced vascular growth and functional recovery [43]. Pharmacological PHD2 inhibition with oridonin further increased OPC HIF-1α stabilization, proliferation, and WNT7A secretion and promoted endothelial angiogenesis through β-catenin-associated signaling [70]. These studies demonstrate that OPC-derived signals can be therapeutically harnessed to promote vascular remodeling, but conditioned-medium delivery does not establish the endogenous sufficiency of resident OPCs, and pharmacological HIF-1α induction should not be interpreted as a constitutive OPC program.

The therapeutic value of angiogenesis is strongly dependent on dose, timing, and vascular maturation. OPC-derived VEGFA and persistent vessel-associated OPC retention can accompany BBB leakage and differentiation arrest in chronic ischemic settings, indicating that greater vascular growth is not inherently reparative. Oligovascular interventions should therefore be evaluated by functional perfusion, vessel stabilization, BBB integrity, and subsequent oligodendroglial lineage progression rather than by vessel density alone. The same principle applies to multimodal agents such as edaravone dexborneol and FGF17: their pleiotropic actions may be therapeutically advantageous, but an OPC contribution should be demonstrated by lineage-resolved target engagement or cell-specific blockade rather than inferred from parallel improvements in imaging, myelin, and behavior.

### Cell transplantation and state-engineered OPC products

OPC transplantation can provide both replacement cells and a concentrated source of paracrine signals. Stereotactic transplantation after transient MCAO reduced acute BBB leakage through WNT7A/β-catenin-associated endothelial signaling [44], while related studies reported increased functional angiogenesis, myelin restoration, and behavioral recovery, with endothelial β-catenin contributing substantially to the white-matter benefit [45]. Delayed transplantation also enhanced endogenous oligodendrogenesis, neurite growth, and synaptogenesis, with CXCR4-associated signaling implicated in endogenous OPC recruitment and Netrin-1/DCC signaling in neuronal effects [11]. More recent work further reported reduced inflammatory cytokines, neutrophil infiltration, and BBB disruption after grafting [125]. These findings support OPC transplantation as a multi-compartment therapeutic strategy rather than a simple cell-replacement approach. However, donor-cell replacement, stimulation of endogenous OPC recruitment or differentiation, and paracrine effects on vascular, immune, or neuronal compartments are distinct mechanisms. Their relative contributions require donor-specific fate mapping, assessment of host-lineage responses, and, where feasible, selective perturbation of the proposed signaling pathway. Improved myelin or behavioral outcomes after transplantation should therefore not be attributed automatically to durable donor-derived remyelination or to an equivalent function of endogenous OPCs.

Preconditioning may allow OPC products to be functionally matched to different phases of repair. Hypoxic conditioning generated severe-hypoxia-associated angiogenic OPC states and milder-hypoxia-associated oligodendrogenic states, and intravenous delivery of the angiogenic population enhanced vascular remodeling and recovery after transient MCAO [69]. This strategy raises the possibility of phase-matched cell products, with angiogenic states used to support earlier vascular repair and oligodendrogenic states potentially reserved for later lineage restoration. However, transcriptomic state labels do not establish stable functional phenotypes after transplantation, and intravenously administered cells may act predominantly through vascular or peripheral paracrine mechanisms rather than durable brain engraftment.

Clinical translation of OPC transplantation will require rigorous definition of cell source, maturation state, potency, dose, delivery route, and release criteria. Stereotactic administration provides direct brain delivery but is invasive, whereas intravenous administration has uncertain brain entry and may rely largely on paracrine mechanisms. Additional concerns include graft survival in ischemic tissue, ectopic differentiation, immunogenicity, batch variability, tumorigenic contamination, vascular obstruction, and difficulty distinguishing donor-cell replacement from stimulation of endogenous repair. Cell-free products such as defined extracellular vesicles may reduce some transplantation-related risks but introduce separate challenges in cargo definition, scalable manufacture, and biodistribution. These barriers currently place OPC transplantation behind pharmacological and rehabilitation-based strategies in clinical readiness despite its broad preclinical efficacy.

### Translational requirements and clinical trial design

No human study has yet demonstrated that an OPC-directed intervention improves stroke outcome through confirmed OPC target engagement and remyelination. The edaravone dexborneol study links a nonrandomized clinical imaging association to parallel mouse and primary-OPC experiments, but does not constitute human mechanistic validation [126]. Similarly, circulating miR-15b-5p correlates with stroke severity and infarct volume, whereas experimental inhibition improves myelin repair through an E2F7/CXCL2-associated pathway [138]; this represents a biomarker–target bridge rather than evidence that serum miR-15b-5p directly reports OPC state. The immediate translational priority is therefore to establish measurable target engagement before testing efficacy in patients selected for demonstrable white-matter or myelin injury.

Clinical endpoints should be matched to the repair transition being tested. Diffusion tensor imaging can identify tract disruption but is not specific for myelin, and should therefore be complemented, where feasible, by validated myelin-sensitive imaging, electrophysiological measures of pathway conduction, blood or cerebrospinal-fluid markers anchored to experimental target engagement, and functional tasks linked to the affected tract. Longitudinal measurements are essential to distinguish acute tissue preservation from delayed remyelination, while peri-infarct and connected remote white matter should be analyzed separately because their repair trajectories may diverge.

Preclinical qualification should test whether therapeutic benefit persists under conditions that better reflect clinical stroke. Candidate interventions should be administered after, rather than only before, ischemia and evaluated across reperfused and nonreperfused models, both sexes, and aged animals with relevant vascular comorbidities such as hypertension or diabetes. Studies should report lesion location and white-matter involvement, confirm exposure within the relevant tissue compartment, and, where feasible, use lineage-restricted loss-of-function approaches to determine whether OPCs are necessary for the observed benefit. Replication across laboratories and comparison with rehabilitation are particularly important for pleiotropic interventions whose behavioral effects may arise from multiple cell populations.

The therapeutic framework that emerges is therefore transition based rather than pathway based. Acute OPC preservation is most relevant when ischemic injury threatens depletion of the viable progenitor pool; promyelinating therapy is most appropriate when recruited OPCs persist but fail to complete differentiation; and niche-directed interventions are warranted when inflammatory, metabolic, vascular, or extracellular-matrix constraints prevent lineage progression. Treatment selection should also account for lesion anatomy: focal white-matter injury may present a different repair bottleneck from large territorial infarction, while peri-infarct and connected remote tracts may require different therapeutic priorities. Age, sex, vascular comorbidities, and reperfusion status should be incorporated into preclinical qualification and, where supported by validated biomarkers, future patient stratification. Cell-based approaches require an additional level of matching between the functional state of the therapeutic product and the biological state of the recipient lesion. Candidate strategies should therefore advance according to their demonstrated ability to move the oligodendroglial lineage through a defined repair bottleneck, rather than by the number of molecular pathways they influence. This transition-based framework links mechanism to treatment timing and provides a practical basis for clinically testable white-matter repair after ischemic stroke.

## Critical knowledge gaps and a translational roadmap

Current evidence establishes OPCs as important contributors to post-stroke white-matter repair, but also reveals a major gap between the mechanistic resolution achievable in experimental systems and that available in patients. Rodent studies can fate-map progenitors, manipulate lineage-specific pathways, and identify newly formed myelin, whereas human studies largely infer OPC behavior from cross-sectional tissue, relative cell abundance, imaging, and circulating biomarkers. Progress will therefore depend less on identifying additional OPC-associated pathways than on resolving lineage flux, validating causal cell states, improving model comparability, and developing clinically measurable indicators of target engagement.

Table 5 translates these uncertainties into a set of decisive experimental priorities. These include reconstructing the human OPC trajectory, determining which reactive states are necessary or sufficient for repair, harmonizing stroke models and lineage endpoints, incorporating age, sex, and vascular comorbidity as biological variables, validating biomarkers against tissue and functional myelin, testing stage-matched treatment sequences, and defining potency, biodistribution, and long-term safety requirements for cell-based products. This roadmap defines both the experiments needed to strengthen causal inference and the boundaries within which current conclusions should remain constrained.

**Table 5:** Critical evidence gaps and decisive studies for translating OPC-centered repair

Priority gap	Current evidence	Decisive next study	Minimum success criterion
Human OPC trajectory	Acute and post-mortem cross-sectional fragments [8, 111, 115]	Time-stratified spatial multi-omics integrated with longitudinal imaging and fluid-biomarker cohorts	Reproducible lesion-compartment state map that constrains plausible lineage transitions
Causal OPC states	Clusters, trajectories, and inferred communication [69, 89, 114]	Inducible lineage-specific perturbation and fate mapping	Demonstrated state necessity or sufficiency for newly formed myelin with a tract-linked functional endpoint
Model comparability	Divergent territorial, focal white-matter, and chronic-ischemia [42, 116, 121, 139]	Common core outcome set across two mechanistically distinct models	Concordant effects on lineage-resolved endpoints across two mechanistically distinct models, with predefined model-specific boundaries
Host heterogeneity	Age, sex, and vascular risks usually tested separately [90, 95, 123, 124]	Factorial aged/comorbid, sex-stratified validation	Defined responder context and safety boundary
Human target engagement	DTI and circulating extracellular vesicles are indirect [126, 140, 141]	Orthogonal imaging, electrophysiology, fluid markers, and treatment-exposure verification	Target-engagement change linked to the predicted repair transition and tract-specific recovery
Treatment sequence	Mostly single target and single time window [3, 133]	Adaptive survival→niche release→differentiation studies	Added benefit over component monotherapies with biomarker-confirmed relief of successive bottlenecks
Cell-product translation	Broad rodent efficacy of grafts and cell-derived products [11, 44, 131, 142]	Standardized potency, biodistribution, and long-term safety	Reproducible product identity, durable safety, and added benefit over resident-cell modulation

### Reconstructing the human OPC trajectory

Human studies currently provide disconnected snapshots of oligodendroglial states rather than a continuous OPC lineage trajectory. Acute single-nucleus sequencing suggests relative enrichment of early OPC states together with depletion of mature oligodendrocytes, but is based on only two stroke samples and three trauma comparators [115]. Post-mortem studies of six stroke cases identify increased OLIG2-positive and early BCAS1-positive cells in peri-infarct cortex and white matter without proportional late-stage BCAS1 accumulation [111], while later lesions contain GPR17-positive/BCAS1-positive differentiation-committed OPCs associated with reactive microglia/macrophages and astrocytic scar formation [8]. These observations are compatible with recruitment followed by incomplete maturation, but cross-sectional human tissue cannot determine the rate, direction, or fate of lineage transitions within an individual lesion. Moreover, none of these studies establishes a causal signaling relationship between inflammatory cells and OPC lineage progression.

Reconstructing the human OPC trajectory will require temporally and spatially resolved tissue sampling rather than larger cross-sectional cohorts alone. Surgical specimens, rapid-autopsy tissue, and archived lesions should be stratified by reperfusion status, lesion compartment, white-matter involvement, age, sex, and major vascular risks. Single-nucleus or spatial transcriptomics should be integrated with multiplexed protein markers that distinguish OPCs, differentiation-committed cells, newly formed oligodendrocytes, and mature myelinating oligodendrocytes. Vascular integrity, axonal preservation, microglial debris-processing states, astrocytic scarring, and extracellular-matrix composition should be mapped within the same spatial coordinates. Although such an approach cannot provide true human lineage tracing, it would substantially constrain the plausible sequence of OPC recruitment, differentiation, arrest, and maturation after stroke.

Longitudinal imaging and fluid biomarkers should be anchored to this tissue-based map of oligodendroglial states. Human stroke lesions show persistent myelin loss and denuded axons, while lineage-specific experiments in mice demonstrate that newly generated myelin can contribute to recovery [3]. The key translational gap is the absence of a validated human signature that distinguishes preservation of pre-existing myelin from de novo remyelination. Prospective cohorts should therefore combine repeated tract-sensitive and myelin-sensitive imaging with electrophysiological measures and biologically anchored fluid markers, and relate these signals to lesion compartment and functional recovery. Tissue datasets can then be used to determine which multimodal signatures most plausibly correspond to OPC recruitment, lineage progression, or remyelination.

### From transcriptomic states to causal OPC functions

Single-cell and spatial studies have substantially expanded the vocabulary of post-stroke OPC states, but state annotation has advanced faster than causal validation. CD44-associated reactive OPCs localize near SPP1-positive myeloid cells, and SPP1 can promote OPC migration in vitro [89]. Spatial profiling further identifies inflammatory-responsive oligodendroglial populations across the infarct core, scar border, and surrounding tissue, while integrated atlases propose severe-hypoxia-associated angiogenic OPC states and milder-hypoxia-associated oligodendrogenic states [69, 114]. These observations provide useful mechanistic hypotheses, but clustering, trajectory inference, spatial proximity, and ligand–receptor prediction do not establish that a state is stable, lineage restricted, necessary, or sufficient for repair.

Establishing causal OPC states will require prospective perturbation rather than retrospective annotation. Candidate state regulators should be manipulated after stroke onset using inducible, lineage-restricted loss- and gain-of-function approaches, followed by fate mapping to determine whether state-defined OPCs return to homeostasis, persist, differentiate, or die. Spatial analysis should test whether the same state has different consequences in the infarct core, peri-infarct tissue, and connected white matter. Functional necessity should then be assessed by newly formed myelin, axonal conduction, neuronal survival, and tract-linked behavior rather than by transcriptomic normalization alone.

Causal direction in cell–cell communication also requires direct testing. Current stroke evidence is stronger for microglial, astrocytic, pericytic, and endothelial regulation of OPC fate than for endogenous OPC control of inflammation, BBB integrity, or synaptic reconstruction, which is often inferred from culture, conditioned-medium, or transplantation studies. Paired sender–receiver perturbations, targeting both signal production in the source cell and the corresponding receptor or downstream response in the recipient cell, would more directly establish endogenous signaling direction and necessity. This approach is particularly important for SPP1, activin A, WNT7A, VEGFA, and extracellular-matrix pathways, whose effects vary with cellular source, concentration, timing, and lesion context.

The distinction between resident microglia and infiltrating MDMs is a particularly important application of this approach. Future studies should combine source-resolved fate mapping with selective manipulation of candidate signals in each myeloid compartment and corresponding receptor or downstream perturbation in OPCs. Such experiments could determine whether MDM-dependent improvements in debris clearance or vascular remodeling are sufficient to account for oligodendroglial recovery, or whether direct MDM–OPC signaling is additionally required [99, 100]. Longitudinal lineage tracing and newly formed myelin, rather than OPC abundance or inferred ligand–receptor interactions alone, should provide the decisive repair endpoints.

### Harmonizing stroke models and repair endpoints

Post-stroke oligodendroglial repair is studied across biologically distinct experimental contexts that should not be treated as interchangeable. Transient and permanent arterial occlusion, photothrombosis, focal white-matter stroke, chronic hypoperfusion, and neonatal hypoxia–ischemia differ in reperfusion, lesion size, gray- versus white-matter involvement, inflammatory milieu, and endogenous repair capacity. Even within transient MCAO, peri-infarct basal ganglia, cortex, and corpus callosum show distinct OPC and oligodendrocyte trajectories [116], while age can alter OPC proliferation and survival after focal white-matter ischemia [139]. Mechanistic claims should therefore remain model-, compartment-, and age-specific unless reproduced across relevant experimental contexts.

A common core outcome set would improve comparability across models without requiring identical experimental designs. Minimum reporting should include lesion location, reperfusion status, white-matter involvement, treatment onset, sex, age, and vascular-risk background. Oligodendroglial outcomes should distinguish OPC abundance, proliferation, migration, differentiation-committed states, newly generated mature oligodendrocytes, and newly synthesized myelin. White-matter repair should additionally be assessed using ultrastructural or validated myelin-sensitive measures, axonal integrity, nodal organization or conduction, and behavior linked to the affected pathway. Infarct-volume reduction should be analyzed separately because smaller lesions may preserve pre-existing myelin without demonstrating active oligodendroglial repair.

Model selection should follow the biological and therapeutic claim being tested. Focal white-matter stroke is well suited to studies of differentiation arrest, remyelination, and tract repair, whereas territorial stroke is required when neuronal loss, BBB disruption, and gray–white matter interactions are central to the proposed mechanism. Chronic hypoperfusion provides a useful model of sustained oligovascular stress but should not be treated as a direct surrogate for acute focal ischemia. Before a pathway is described as a general post-stroke OPC mechanism, its key effects should ideally be reproduced in at least one mechanistically distinct stroke model.

### Age, sex, and vascular comorbidity as biological variables

Age, sex, and vascular comorbidity can alter the biological mechanism of oligodendroglial repair rather than simply increase injury severity. Aging and hypertension reduce early OPC abundance and proliferative responses after ischemia [123], while diabetes impairs oligodendrogenesis, white-matter conduction, and reparative microglial support [124]. Hyperhomocysteinemia introduces a TNF-α/PAD4-associated block to OPC differentiation [90], and microglial TNFR2 exerts sex-dependent effects on myelin-debris clearance and oligodendrocyte maturation [95]. These observations indicate that the same therapeutic target may operate differently across biological and vascular-risk backgrounds.

Preclinical qualification should therefore incorporate age, sex, and clinically relevant vascular risk as interacting biological variables rather than as token additions to otherwise homogeneous designs. Factorial approaches can determine whether these factors modify treatment timing, target engagement, or the specific repair transition being addressed, including OPC preservation, release from differentiation arrest, and restoration of supportive immune states. When therapeutic benefit is restricted to a defined biological context, clinical translation should favor biomarker-enriched enrollment rather than dilute the effect in an unselected stroke population. A context-specific treatment response should therefore be regarded as biologically informative rather than as evidence that the therapeutic strategy has failed to generalize.

### Biomarkers, target engagement, and patient selection

A clinical OPC program requires biomarkers that identify both an available white-matter repair substrate and the lineage transition targeted by treatment. Diffusion tensor imaging can localize tract disruption but is not specific for myelin or OPC activity. Circulating extracellular vesicles may provide a complementary molecular readout: presumed CD9-positive/MOG-positive oligodendrocyte-derived vesicles have been measured longitudinally in 58 patients after stroke, while a separate study detected changes in oligodendrocyte-derived vesicles in 14 ischemic stroke cases [140, 141]. However, their relationship to lesion burden, OPC state, and remyelination remains uncertain, and MOG-positive vesicles are more likely to reflect differentiated oligodendroglial or myelin biology than OPC abundance. These assays should therefore be regarded as candidate white-matter or oligodendroglial biomarkers rather than validated OPC markers. Validation should establish cell of origin and test whether longitudinal changes track myelin-sensitive imaging, electrophysiology, lesion compartment, and functional recovery while distinguishing acute tissue destruction from delayed repair.

Early-phase trials should enroll a biologically selected population, confirm treatment exposure, prespecify an OPC- or myelin-related target-engagement signature, and test whether changes in that intermediate endpoint predict tract-specific functional recovery. Clinical scales remain essential for evaluating patient benefit, but should be complemented by mechanistic readouts capable of linking treatment to the proposed repair process.

### Sequential and combination repair strategies

Because post-stroke oligodendroglial repair is constrained by serial biological bottlenecks, staged combination therapy may be more rational than stronger stimulation of a single pathway. Acute cytoprotection could preserve the viable OPC pool, a subsequent niche-directed intervention could improve myelin-debris clearance and relieve inhibitory extracellular-matrix or inflammatory constraints, and later promyelinating therapy could drive lineage completion. The sequence is likely to matter: premature differentiation may deplete or misdirect progenitors in unstable tissue, whereas prolonged proliferative stimulation may expand an immature reactive population without restoring myelin. Combination strategies should therefore be organized around successive repair transitions rather than simultaneous polypharmacy.

Adaptive preclinical designs could test these staged strategies more rigorously by advancing treatment only when a prespecified marker indicates that the preceding repair bottleneck has been sufficiently relieved. For example, promyelinating therapy could be initiated only after confirming preservation of an adequate OPC pool, whereas differentiation-promoting treatment might be delayed until myelin-debris clearance has reached a defined threshold. Reciprocal OPC-specific *Olig2* and *Chrm1* manipulations establish lineage completion as a causal determinant of recovery [3], while delayed cGAS–STING inhibition demonstrates that niche modification can remain effective beyond the acute phase [133]. These findings provide a mechanistic basis for biomarker-guided, sequential intervention rather than indiscriminate combination therapy.

Cell-based products should follow the same stage-matching principle. Human induced pluripotent stem cell (iPSC)-derived OLIG2-programmed OPCs have promoted remyelination and functional recovery after transplantation in rat transient MCAO, but product identity, potency, biodistribution, and long-term safety remain preclinical concerns [142]. State-engineered grafts and extracellular-vesicle products should therefore be matched to lesion phase and compared directly with strategies that modulate endogenous repair. Their added manufacturing, delivery, and safety complexity is justified only if they provide a reparative function that cannot be achieved more safely through pharmacological treatment, rehabilitation, or manipulation of resident cells.

## Conclusions

OPCs integrate neural activity, myelin homeostasis, inflammatory, metabolic, and vascular signals, and ischemic stroke disrupts this integration in a spatially and temporally heterogeneous manner. Severe ischemia depletes the progenitor pool, whereas partially perfused tissue permits reactive recruitment and state divergence within neuroimmune–oligovascular niches. Long-term repair depends not on OPC accumulation alone but on whether recruited cells complete differentiation and generate stable, functional myelin. OPC accumulation and remyelination failure are therefore compatible observations rather than opposing conclusions.

The strongest evidence for a beneficial OPC function after stroke is the causal contribution of lineage completion and newly formed myelin to neuronal survival and functional recovery. By contrast, endogenous OPC regulation of angiogenesis, BBB integrity, inflammation, and synaptic reconstruction remains less firmly established and often relies on conditioned-medium experiments, pharmacological induction, or transplantation. Therapeutic interpretation should preserve this evidence hierarchy: increasing precursor abundance is not sufficient, and effective repair ultimately requires appropriate recruitment, release from differentiation arrest, generation of mature oligodendrocytes, formation of functional myelin, and preservation of the repaired tract.

A clinically meaningful OPC strategy will need to be phase-matched, compartment-aware, and biologically stratified. Progress will require integration of lineage-resolved experimental causality with human spatial evidence, validated target-engagement biomarkers, myelin- and tract-specific outcomes, and testing in aged, sex-balanced, comorbid models. OPC-centered repair should therefore be viewed as a testable component of post-stroke reconstruction rather than a generalized regenerative principle. Its clinical value will ultimately depend on demonstrating when, where, and through which lineage transition OPC-centered intervention changes human recovery.

## Data Availability

No datasets were generated or analysed during the current study.

## Abbreviations

AA: Arachidonic acid
ACSL4: Acyl-CoA synthetase long-chain family member 4
ACSS2: Acyl-CoA synthetase short-chain family member 2
ALDOC: Aldolase C
AMPA: α-amino-3-hydroxy-5-methyl-4-isoxazolepropionic acid
AMPK: AMP-activated protein kinase
APC: Adenomatous polyposis coli
APAF-1: Apoptotic peptidase-activating factor 1
ARE: Antioxidant response element
ASC: Apoptosis-associated speck-like protein containing a CARD
ATP: Adenosine triphosphate
BBB: Blood–brain barrier
BCAS1: Breast carcinoma amplified sequence 1
BNIP3: BCL2-interacting protein 3
CaMK: Ca^2+^/calmodulin-dependent protein kinase
CCN3: Cellular communication network factor 3
CCR2: C-C chemokine receptor type 2
cGAS: Cyclic GMP–AMP synthase
CHOP: C/EBP homologous protein
CK2: Casein kinase 2
CNP: 2′,3′-cyclic nucleotide 3′-phosphodiesterase
CNS: Central nervous system
cPLA2α: Cytosolic phospholipase A2α
CREB: CAMP-response element-binding protein
CSPG4: Chondroitin sulfate proteoglycan 4
CUL3: Cullin 3
DTI: Ciffusion tensor imaging
ECM: Extracellular matrix
EGFR: Epidermal growth factor receptor
ENPP6: Ectonucleotide pyrophosphatase/phosphodiesterase 6
ERK: Extracellular signal-regulated kinase
EV: Extracellular vesicle
FGF17: Fibroblast growth factor 17
GPX4: Glutathione peroxidase 4
GPR17: G protein-coupled receptor 17
GRP78/BiP: 78-kDa glucose-regulated protein/binding immunoglobulin protein
GSDMD: Gasdermin D
GSH: Reduced glutathione
HDAC3: Histone deacetylase 3
HIF-1α: Hypoxia-inducible factor 1α
HSP90α: Heat shock protein 90α
HPβCD: Hydroxypropyl-β-cyclodextrin
IFN-γ: Interferon-γ
IL-1β: Interleukin-1β
IL-18: Interleukin-18
iPSC: Induced pluripotent stem cell
JNK: C-Jun N-terminal kinase
JNK3: C-Jun N-terminal kinase 3
Keap1: Kelch-like ECH-associated protein 1
LAL: Lysosomal acid lipase
LC3: Microtubule-associated protein 1 light chain 3
LCN2: Lipocalin 2
LINGO-1: Leucine-rich repeat and immunoglobulin domain-containing Nogo receptor-interacting protein 1
LPCAT3: Lysophosphatidylcholine acyltransferase 3
LRP2: Low-density lipoprotein receptor-related protein 2
MAG: Myelin-associated glycoprotein
MBP: Myelin basic protein
MCAO: Middle cerebral artery occlusion
MCT1: Monocarboxylate transporter 1
MDM: Monocyte-derived macrophage
MFSD2A: Major facilitator superfamily domain-containing protein 2 A
MHC: Major histocompatibility complex
miR-219: MicroRNA-219
MLKL: Mixed-lineage kinase domain-like protein
MOG: Myelin oligodendrocyte glycoprotein
MYRF: Myelin regulatory factor
mPTP: Mitochondrial permeability transition pore
mtDNA: Mitochondrial DNA
NADPH: Nicotinamide adenine dinucleotide phosphate
Nec-1: Necrostatin-1
NG2: Neural/glial antigen 2
NLRP3: NLR family pyrin domain containing 3
NMDAR: N-methyl-D-aspartate receptor
nNOS: Neuronal nitric oxide synthase
Nrf2: Nuclear factor erythroid 2-related factor 2
NVU: Neurovascular unit
OGD: Oxygen–glucose deprivation
OGD/R: Oxygen–glucose deprivation/reoxygenation
OL: Oligodendrocyte
OLIG2: Oligodendrocyte transcription factor 2
OPC: Oligodendrocyte precursor cell
PAD4: Peptidylarginine deiminase 4
PDGFRα: Platelet-derived growth factor receptor-α
PHD2: Prolyl hydroxylase domain-containing protein 2
PHGDH: Phosphoglycerate dehydrogenase
PI3K: Phosphoinositide 3-kinase
PINK1: PTEN-induced kinase 1
PL: Phospholipid
PLIN2: Perilipin 2
PLP1: Proteolipid protein 1
PRMT5: Protein arginine methyltransferase 5
PSD95: Postsynaptic density protein 95
RIPK1: Receptor-interacting serine/threonine-protein kinase 1
RIPK3: Receptor-interacting serine/threonine-protein kinase 3
ROS: Reactive oxygen species
SHP2: Src homology 2 domain-containing protein tyrosine phosphatase 2
SIK3: Salt-inducible kinase 3
SLC11A1: Solute carrier family 11 member 1
SOD2: Superoxide dismutase 2
SOX10: SRY-box transcription factor 10
SPP1: Secreted phosphoprotein 1
STING: Stimulator of interferon genes
SVZ: Subventricular zone
tMCAO: Transient middle cerebral artery occlusion
TGF-α: Transforming growth factor-α
TGF-β: Transforming growth factor-β
TNF-α: Tumor necrosis factor-α
TNFR2: Tumor necrosis factor receptor 2
Treg: Regulatory T cell
TREM2: Triggering receptor expressed on myeloid cells 2
TRPM2: Transient receptor potential melastatin 2
TRX: Thioredoxin
TUNEL: Terminal deoxynucleotidyl transferase dUTP nick-end labeling
TXNIP: Thioredoxin-interacting protein
Ub: Ubiquitin
VEGFA: Vascular endothelial growth factor A
XBP1: X-box binding protein 1
